# Efficacy and safety of isotonic versus hypotonic intravenous maintenance fluids in hospitalized children: an updated systematic review and meta-analysis of randomized controlled trials

**DOI:** 10.1007/s00467-023-06032-7

**Published:** 2023-06-26

**Authors:** Basma Ehab Amer, Omar Ahmed Abdelwahab, Ahmed Abdelaziz, Youssef Soliman, Ahmed Mostafa Amin, Maged Ahmed Mohamed, Khaled Albakri, Esraa Mohamed Zedan, Naema Hamouda

**Affiliations:** 1 Medical Research Group of Egypt, Cairo, Egypt; 2 Faculty of Medicine, Benha University, Benha, Egypt; 3 Faculty of Medicine, Al-Azhar University, Cairo, Egypt; 4 Faculty of Medicine, Assiut University, Assiut, Egypt; 5 Faculty of Dentistry, Al-Azhar University, Cairo, Egypt; 6 Faculty of Medicine, The Hashemite University, Zarqa, Jordan; 7 General Organization of Teaching Hospitals and Institutes, Cairo, Egypt

**Keywords:** Intravenous fluid, Isotonic, Hypotonic, Hyponatremia, Hypernatremia, Children

## Abstract

**Background:**

Iatrogenic hyponatremia is a common complication following intravenous maintenance fluid therapy (IV-MFT) in hospitalized children. Despite the American Academy of Pediatrics' 2018 recommendations, IV-MFT prescribing practices still vary considerably.

**Objectives:**

This meta-analysis aimed to compare the safety and efficacy of isotonic versus hypotonic IV-MFT in hospitalized children.

**Data sources:**

We searched PubMed, Scopus, Web of Science, and Cochrane Central from inception to October 1, 2022.

**Study eligibility criteria:**

We included randomized controlled trials (RCTs) comparing isotonic versus hypotonic IV-MFT in hospitalized children, either with medical or surgical conditions. Our primary outcome was hyponatremia following IV-MFT. Secondary outcomes included hypernatremia, serum sodium, serum potassium, serum osmolarity, blood pH, blood sugar, serum creatinine, serum chloride, urinary sodium, length of hospital stay, and adverse outcomes.

**Study appraisal and synthesis methods:**

Random-effects models were used to pool the extracted data. We performed our analysis based on the duration of fluid administration (i.e., ≤ 24 and > 24 h). The Grades of Recommendations Assessment Development and Evaluation (GRADE) scale was used to evaluate the strength and level of evidence for recommendations.

**Results:**

A total of 33 RCTs, comprising 5049 patients were included. Isotonic IV-MFT significantly reduced the risk of mild hyponatremia at both ≤ 24 h (RR = 0.38, 95% CI [0.30, 0.48], *P* < 0.00001; high quality of evidence) and > 24 h (RR = 0.47, 95% CI [0.37, 0.62], *P* < 0.00001; high quality of evidence). This protective effect of isotonic fluid was maintained in most examined subgroups. Isotonic IV-MFT significantly increased the risk of hypernatremia in neonates (RR = 3.74, 95% CI [1.42, 9.85], *P* = 0.008). In addition, it significantly increased serum creatinine at ≤ 24 h (MD = 0.89, 95% CI [0.84, 0.94], *P* < 0.00001) and decreased blood pH (MD = –0.05, 95% CI [–0.08 to –0.02], *P* = 0.0006). Mean serum sodium, serum osmolarity, and serum chloride were lower in the hypotonic group at ≤ 24 h. The two fluids were comparable in terms of serum potassium, length of hospital stay, blood sugar, and the risk of adverse outcomes.

**Limitations:**

The main limitation of our study was the heterogeneity of the included studies.

**Conclusions and implications of key findings:**

Isotonic IV-MFT was superior to the hypotonic one in reducing the risk of iatrogenic hyponatremia in hospitalized children. However, it increases the risk of hypernatremia in neonates and may lead to renal dysfunction. Given that the risk of hypernatremia is not important even in the neonates, we propose to use balanced isotonic IV-MFT in hospitalized children as it is better tolerated by the kidneys than 0.9% saline.

**Systematic review registration number:**

CRD42022372359.

**Graphical abstract:**
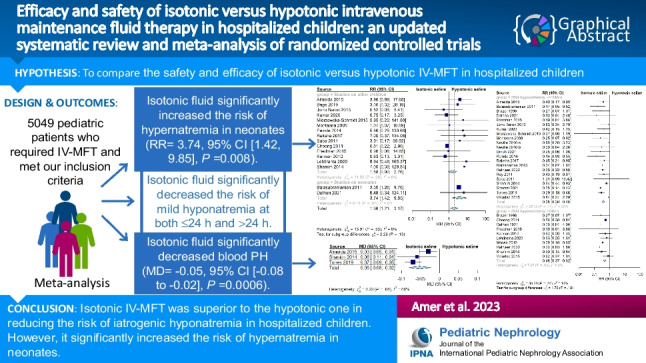
A higher resolution version of the Graphical abstract is available as Supplementary information

**Supplementary Information:**

The online version contains supplementary material available at 10.1007/s00467-023-06032-7.

## Introduction

Physicians prescribe maintenance fluid therapy for otherwise healthy hospitalized children to preserve their extracellular volume and electrolyte balance [1]. It can be provided through enteral or intravenous routes. However, intravenous maintenance fluid therapy (IV-MFT) is the standard of care for many hospitalized children who cannot maintain their fluid requirements through enteral intake. Variable preparations of intravenous fluid are available. They are either isotonic or hypotonic solutions relative to plasma. Normal saline, ringer lactate, and acetate are examples of isotonic solutions that are close to plasma osmolality (270 to 310 mOsm/L), while 0.45% and 0.18% normal saline are examples of hypotonic ones [2, 3]. The wide variability of maintenance fluids makes it challenging for physicians to determine the best IV-MFT for their patients.

For decades, the maintenance fluid therapy was calculated according to the Holiday–Segar method, in which 0.25% normal saline was used [4]. However, Arieff and Fraser described the occurrence of iatrogenic hyponatremia in generally healthy children who received a hypotonic solution with a sodium content of 38 mmol/l. Hyponatremia was severe enough to cause death and permanent brain damage for survivors [5]. Since then, many studies have been conducted on children requiring IV-MFT. Most of which revealed evidence that the hypotonic solution is associated with a higher risk of iatrogenic hyponatremia [6–9].

The American Academy of Pediatrics and the National Institute for Health and Care Excellence (NICE) recommended using the isotonic solution as IV-MFT for children in 2018 and 2020, respectively [10, 11]. Despite these recommendations, IV-MFT prescribing practices vary considerably [12, 13]. This gap between prescribing practice and what should be used necessitates the availability and dissemination of clear evidence-based guidelines.

Due to the inconsistent results of the published RCTs about the preferable IV-MFT, multiple meta-analyses have been conducted. These meta-analyses have pointed out that isotonic fluid would be a safer choice for IV-MFT in children as there was a significantly higher risk of hyponatremia following the hypotonic fluid [14–20]. However, there are eleven randomized controlled trials (RCTs) that were not included in the last meta-analysis by Hasim et al. Interestingly, three of these RCTs [21–23] revealed no difference between isotonic and hypotonic IV-MFT while the remaining RCTs [24–31] favored the isotonic one. Therefore, we conducted this systematic review and meta-analysis to provide updated evidence from all published randomized clinical trials and compare the safety and efficacy of isotonic versus hypotonic IV-MFT in hospitalized children.

## Methods

We conducted our systematic review and meta-analysis according to the Preferred Reporting Items for Systematic Review and Meta-Analysis (PRISMA) guidelines and the Cochrane Handbook of Systematic Reviews for interventions [32, 33]. Our study protocol is registered in the PROSPERO database (registration number: CRD42022372359).

## Literature search and data collection

On October 1, 2022, we performed an electronic search in four databases: PubMed, Scopus, Web of Science, and Cochrane Central. We used the following keywords: isotonic, hypotonic, saline, NaCl, pediatric, newborn, and hyponatremia. We summarized the search strategy for each database in detail in Supplementary Table 1. The search was carried out by two authors (B.E. and O.A.) independently. Additionally, we reviewed the reference list of included studies and relevant systematic reviews for any missing eligible RCTs. We then removed duplicates using both the EndNote X8 program and Rayyan [34].

## Study selection and eligibility criteria

We included RCTs comparing isotonic versus hypotonic IV-MFT in hospitalized children with either medical or surgical conditions. Our primary outcome was hyponatremia, including mild hyponatremia (defined as serum sodium < 135 mmol/L), moderate hyponatremia (defined as serum sodium < 130 mmol/L), and severe hyponatremia (defined as serum sodium < 125 mmol/L) at any time while receiving IV-MFT. Secondary outcomes were hypernatremia (defined as serum sodium > 145 mmol/L), serum sodium, serum potassium, serum osmolarity, blood pH, blood sugar, serum creatinine, serum chloride, urinary sodium, length of hospital stay, and adverse outcomes. An isotonic fluid is defined as any fluid with an osmolality equal to that of plasma, such as normal saline (0.9% sodium chloride), Ringer's lactate, or Hartmann's solution. Hypotonic fluid is defined as any fluid with an osmolarity lower than that of 0.9% sodium chloride, such as 0.18%, 0.3%, or 0.45% sodium chloride. We excluded studies that involved patients with abnormal baseline serum sodium.

Three independent authors (A.M., E.M., and N.H.) screened the articles for eligibility in two steps: the title and abstract screening using Rayyan and the full-text screening. They consulted a fourth author (B.E.) to discuss and resolve any conflicts or disagreements.

## Methodological quality assessment

At least two authors (A.M. and E.M.) or (M.A., and N.H.) independently assessed the quality of each trial using the Risk of Bias Assessment tool-2 (ROB2) [35]. Disagreements were resolved through a discussion with a third author (B.E.). The ROB2 tool involves the following five domains: randomization process, deviations from intended interventions, measurement of the outcome, missing outcome data, selection of the reported results, and other biases. The overall authors' judgment for each domain fell into three categories: low, some concerns, and high risk of bias.

The Grades of Recommendations Assessment Development and Evaluation (GRADE) scale was used to evaluate the strength and level of evidence for recommendations and was stratified as follows: high quality, which indicates no further research is needed and is unlikely to change the confidence in the effect estimations; moderate quality, which indicates that further studies may affect the confidence in the effect estimation; low quality, which indicates further research is likely to have a crucial impact on the confidence in the effect estimate and may change the estimate; and very low quality, which indicates that we are not certain about this estimate.

## Data extraction

At least two authors (A.M. and E.M.) or (M.A. and N.H.) independently extracted the data of interest. They consulted another author (B.E.) or (O.A.) to discuss any disagreement. In a Google Sheet, we extracted the following data: (1) characteristics of the included studies and populations, (2) risk of bias assessment, and (3) primary and secondary outcomes. All outcomes were documented at the time points reported in the study (at 2, 4, 6, 8, 12, 16, 18, 24, 36, 48, 72 hours, and 7 days). We then pooled these outcomes at ≤ 24 and > 24 h because most studies had outcome measurements at 24 h.

## Data analysis

We conducted all the analyses and plots using RStudio with the meta package. We used the metacont function to analyze continuous variables, and the metabin function to analyze categorical outcomes. We carried out our analysis using the random effect model. We preferred this model because, in contrast to the fixed effects one, it allows for a higher standard error in the pooled estimate and makes it appropriate for controversial or inconsistent estimates. In addition, this model gives smaller studies a somewhat higher weight than larger studies and assumes that the included studies represent a random sample from the population. We used risk ratio (RR) with a 95% confidence interval and the Mantel–Haenszel method to estimate dichotomous outcomes, while mean difference (MD) with a 95% confidence interval and the inverse variance was used for continuous outcomes. We used the I^2^ and Chi-square p-value to assess significant levels of heterogeneity.

We performed our analysis based on the duration of fluid administration (i.e., ≤ 24 and > 24 h) on all the primary and secondary outcomes except length of hospital stay, blood pH, blood sugar, urine sodium, and adverse events because they were reported in only a few studies. For studies that measured the outcome at multiple time points, we selected the nearest point to 24 h to be included in the ≤ 24 h group, while those measurements at the endpoint were selected for the > 24 h group to avoid duplication. In addition, we conducted subgroup analyses on mild, moderate, and severe hyponatremia at each time point. Moreover, we performed subgroup analysis on mild hyponatremia based on the condition of hospitalized children (surgical versus medical), the sodium concentration of the hypotonic fluid (moderately hypotonic versus very hypotonic), the rate of fluid administration (maintenance rate versus restricted rate), the blinding of study personnel (open label studies versus blinded studies), and different regions of the included studies. We included fluids with 0.45% sodium chloride in the moderately hypotonic group, while those with lower sodium chloride concentrations were included in the very hypotonic one. Regarding the rate of IV-MFT, we included rates ≤ 70% of the standard maintenance rate in the restricted group, while those ranging from 80 to 120% were included in the maintenance group, as recommended by Holliday 1957 [4]. Furthermore, we performed subgroup analysis on hypernatremia and mild hyponatremia based on the age category (neonates versus other children). Finally, we conducted subgroup analyses on mild hyponatremia and serum creatinine based on the composition of isotonic solutions (balanced versus 0.9% saline). Ringer’s lactate, Hartman's solution, Ringer's acetate, and Plasma-Lyte were included in the balanced fluids subgroup.

## Results

### Literature search results

**Fig. 1:**
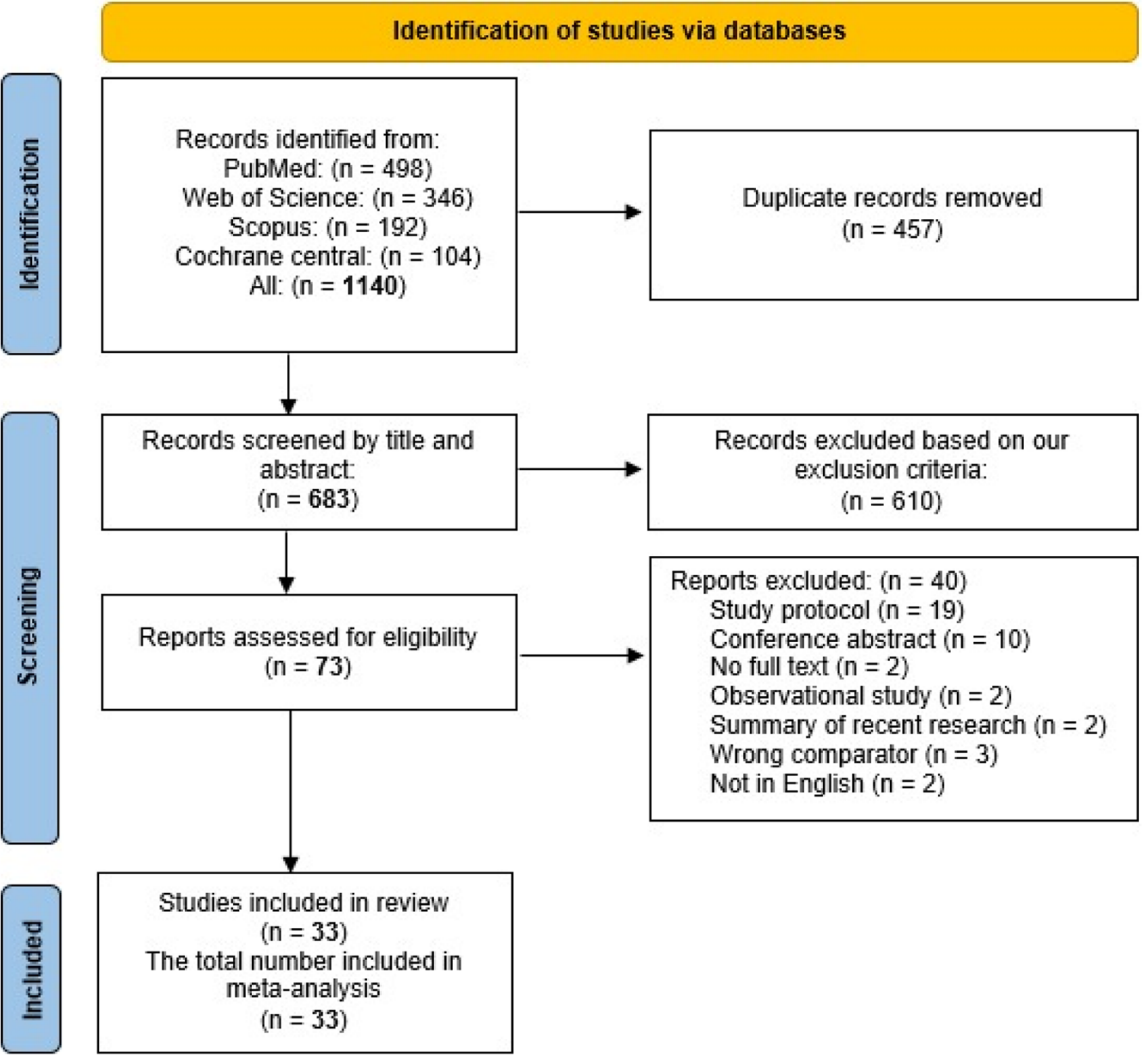
The PRISMA flow diagram

Our search identified 1140 records for screening. After removing duplicates, 683 articles remained for the title and abstract screening. We excluded 610 articles, while only 73 seemed to be eligible. After reading the full text of the 73 studies, we included only 33 eligible studies in the qualitative and quantitative synthesis. Figure 1 shows the PRISMA flow diagram for study selection.

### Study characteristics

This meta-analysis included 5049 children hospitalized for various medical and surgical conditions. All studies were RCTs conducted in 13 different countries: India (n = 11), Australia (n = 6), Canada (n = 3), Spain (n = 2), Nigeria (n = 2), Argentina (n = 2), Portugal (n = 1), Mexico (n = 1), Finland (n = 1), Poland (n = 1), Iran (n = 1), Brazil (n = 1), and Pakistan (n = 1). The duration of follow-up ranged from two hours to seven days. Shatabi et al. 2022 and Mierzewska-Schmidt et al. 2015 had the shortest follow-up period, while the longest one was in Lehtiranta et al. 2021 [28, 36, 37]. We provide a summary of the included studies and baseline characteristics of patients in Tables 1 and Supplementary Table 2, respectively. Two studies (Neville et al. 2010 and Yung et al. 2009) were four-arm studies. They both had two arms for the isotonic fluid and another two arms for the hypotonic one. Each of the fluid arms were similar in their sodium concentrations but different in their rates of administration. In our meta-analysis, we pooled the data for each rate separately and treated each study as two separate studies, denoted in our forest plot as Neville 2010 a, Neville 2010 b, Yung 2009 a, and Yung 2009 b. According to the ROB-2 tool, the quality of included studies ranged from high to low. Twelve (36.4%) of the studies had a low risk of bias; thirteen (39.4%) were considered as having some concerns, while the remaining studies (24.2%) had a high risk of bias. The risk of bias summary and graph are shown in Supplementary Fig.1A and Supplementary Fig. 1B, respectively.

**Table 1:** summary of the included studies

Study ID	Country	RCT design		Timepoint of reporting outcomes (hours)	Participants	Groups	Sample size	Main outcomes reported	Key findings
		Blinded or open-label	Single or multicenter						
Almeida 2015 [38]	Portugal	Open-label	Single	24	Patients from 1 day to 18 years old with an acute injury (medical or surgical) who required intravenous fluid therapy for longer than 24 h	0.9% NaCl in 5% dextrose	130	The change in sodium levels between T0 and T24 hours with maintenance fluids A and B, the occurrence of hyponatremia or hypernatremia, bicarbonate, pH, base excess, urine excretion of sodium and chloride, and tCO2 and chloride at T24 hours	Normal saline significantly decreased the risk of hyponatremia and hypernatremia
						0.45% NaCl in 5% dextrose	103		
Bagri 2019 [39]	India	Open-label	Single	24 and 48	Children at the age of 1 month and 18 years who required to maintain intravenous fluids for a variety of medical conditions while they were hospitalized	0.9% NaCl in 5% dextrose at a standard maintenance rate	75	Hyponatremia and hypernatremia at 24 and 48 h of hospital admission, seizures, encephalopathy, weight gain, and edema	The incidence of hyponatremia did not significantly differ between the two groups
						0.45% NaCl in 5% dextrose at a standard maintenance rate	75		
Balasubramanian 2011 [40]	India	Blinded	Single	8 and 24	Full-term newborn babies who had severe non-hemolytic hyperbilirubinemia	0.9% saline in 5% dextrose at a standard maintenance rate	42	The number of newborns who developed hyponatremia after 8 h of IV fluid therapy, the number of infants who received BET, the duration of phototherapy, the number of infants who developed hypernatremia, the rate of change in STB, and plasma sodium	Hypotonic fluid increased the incidence of hyponatremia, while the isotonic one increased the risk of hypernatremia
						0.2 saline in 5% dextrose at standard maintenance rate	42		
Brazel 1996 [41]	Australia	NR	Single	6, 24, 48, and 72	Patients were undergoing primary corrective surgery for idiopathic scoliosis	Isotonic saline (Hartman's solution) at a standard maintenance rate	5	The development of SIADH after surgery is defined by hyponatremia and serum hypo-osmolarity	Hypotonic fluid was associated with the development of SIADH. Isotonic saline should be used
						0.3 normal saline in 3% dextrose or 0.18 normal saline in 4% dextrose at a standard maintenance rate	7		
Chinnasami 2022 [21]	India	Open-label	Single	48	Children from 3 months to 12 years old who were considered by the treating physician to need Intravenous maintenance fluid therapy for at least 48 h	0.9% saline in 5% dextrose at a standard maintenance rate	50	Urea, creatinine, the difference in change in sodium, potassium, chloride, and bicarbonate among the three IV fluids, adverse events like dehydration, shock, over-hydration, seizures, and headache after 48 h	The three groups were not significantly different from each other
						0.18% saline in 5% dextrose at a standard maintenance rate	50		
						Isotonic saline (Plasma-Lyte 148) at a standard maintenance rate	50		
Choong 2011 [42]	Canada	Blinded	Single	24 and 48	Patients undergoing surgery between the ages of 6 months and 16 years with a postoperative stay exceeding 24 h	0.9% saline in 5% dextrose	128	Hyponatremia, severe hyponatremia, hypernatremia, plasma ADH levels, adverse reactions that occurred within 48 h of the intervention, and the percentage of patients who switched to open-label PMS therapy during the trial	Isotonic saline significantly decreased the risk of postoperative hyponatremia
						0.45% saline in 5% dextrose	130		
Coulthard 2012 [43]	Australia	Open-label	Single	16–18	Children who were scheduled for spinal instrumentation or craniotomy to remove their brain tumors or remodel their cranial vault	Isotonic saline (Hartman's solution) at a standard maintenance rate	41	Plasma sodium at 16–18 h after surgery and the total number of fluid boluses given	Hartman's solution caused less decrease in postoperative plasma sodium
						0.45% saline in 5% dextrose at 2/3 of a standard rate	41		
Dathan 2021 [25]	India	Triple-blinded	Single	24, 48, and 72	Neonates who are at least 34 weeks gestational age, 48 h postpartum, and 28 days old. They were euvolemic with normal initial serum sodium, serum potassium, and serum glucose levels. They only required IV maintenance fluid after an intravenous bolus of an isotonic fluid prior to enrolment	0.9% NaCl in 5% dextrose	31	Hyponatremia and hypernatremia at 24, 48, and 72 h, mean serum osmolarity at the end of the trial, edema, and weight change throughout the study	The difference between isotonic and hypotonic saline was insignificant in terms of developing hyponatremia at 24 h; however, there was a highly significant difference favoring the isotonic saline in terms of developing hypernatremia at 48 h
						0.15% NaCl in 5% dextrose	29		
Flores Robles 2015 [44]	Mexico	Blinded	Single	8	Children with acute medical or surgical disorders, aged 3 months to 15 years, who were brought into the emergency room and needed IV fluid therapy for at least 8 h	0.9% saline in 5% dextrose	52	Serum sodium levels, hospital-acquired hypo or hypernatremia at 8 h, moderate or severe hyponatremia, headache, vomiting, fever, seizures, edema, hypertension, dehydration, and duration of hospital stay	Isotonic solutions were superior to hypotonic ones in reducing hospital-acquired hyponatremia
						0.45% saline in 5% dextrose	50		
						0.3% saline in 5% dextrose	49		
Friedman 2015 [7]	Canada	Double-blinded	Single	24 and 48	Children from 1 month to 18 years old with normal baseline serum sodium levels who needed IV fluids for at least 48 h without further rehydration after enrolment	0.9% saline in 5% dextrose at a standard maintenance rate	54	Mean serum sodium level at 24 and 48 h, hyponatremia, hypernatremia, weight gain, hypertension, and edema	Isotonic maintenance fluid was safe and might be superior to hypotonic maintenance fluid in reducing hyponatremia in pediatric patients
						0.45% saline in 5% dextrose at a standard maintenance rate	56		
Jorro Baron 2013 [45]	Argentina	Double-blinded	Single	24	Children aged 1 month to 18 years with normal initial serum sodium who required > 80% of the total IV maintenance fluids and were expected to be in the PICU for more than 24 h	0.9% saline in 5% dextrose at 77% of the standard maintenance rate	31	Hyponatremia, hypernatremia, severe hyponatremia, serum Na change at 24 h, 28-day mortality following randomization, days without mechanical ventilation since PICU admission, and the length of PICU stay	Between hypotonic and isotonic fluids, there was no difference in terms of developing iatrogenic hyponatremia at 24 h
						0.45% saline in 5% dextrose at 63% of the standard maintenance rate	32		
Raksha 2017 [6]	India	Open-label	Single	24	Children in the PICU whose conditions were determined by the attending physician to require IV fluids for at least 24 h	0.9% saline in 5% dextrose at a standard maintenance rate	120	Hyponatremia, hypernatremia, symptomatic hyponatremia or hypernatremia, duration of ICU stay, improvement, and death	Isotonic saline was superior to hypotonic saline in reducing acquired hyponatremia and ICU admission among children
						0.18% saline in 5% dextrose at a two-third standard maintenance rate	120		
Ramanathan 2015 [46]	India	Open-label	Single	6, 12, and 24	All euvolemic children in the general pediatric ward between the ages of 2 months and 5 years who needed parenteral maintenance fluids and met the WHO clinical diagnosis of very severe pneumonia	0.9% saline in 5% dextrose at a standard maintenance rate	59	Mild, moderate, and severe hyponatremia, hypernatremia, lethargy, gait disturbances, signs of anisocoria, papilledema, cardiac arrhythmias, and coma	The study favored the use of isotonic PMS in children with respiratory infections
						0.18% saline in 5% dextrose at a standard maintenance rate	60		
Ratnjet 2022 [27]	India	Open-label	Single	12, 24, and 48	Hospitalized children between the ages of 3 months and 12 years who needed IV fluid therapy	0.9% saline in 5% dextrose	100	Alterations in initial serum sodium levels after 24 and 48 h, hyponatremia, and hypernatremia	Hospitalized children were more likely to develop hyponatremia when given hypotonic fluids
						0.45% saline in 5% dextrose	100		
Rey 2011 [29]	Spain	NR	Multicenter	12 and 24	Patients in the PICUs who required IV maintenance fluid therapy	Isotonic fluids (156 mmol/L tonicity)	63	Serum sodium at 12 and 24 h, hyponatremia, and hypernatremia	Hyponatremia occurred more frequently with the hypotonic solutions
						Hypotonic fluids (50–70 mmol/L tonicity)	62		
Saba 2011 [8]	Canada	Double-blind	Single	12	Children aged 3 months to 18 years who needed a minimum of 8 h of IV fluids for elective surgeries or following their admission to the emergency department	0.9% saline in 5% dextrose at a mean rate = 109.03% of the standard maintenance rate	16	Hyponatremia, hypernatremia, absolute change in sodium, sodium change rate, hypertension, and adverse events related to hyponatremia	Hypotonic saline did not decrease serum Na level at 12 h in children without severe baseline hyponatremia
						0.45% saline in 5% dextrose at a mean rate = 113.6% of the standard maintenance rate	21		
Kannan 2010 [47]	India	Open-label	Single	12, 24, 48, and 72	Children who needed IV-MFT for at least 24 h and ranged in age from 3 months to 12 years	0.9% saline in 5% dextrose at a standard maintenance rate	58	Hyponatremia, symptomatic hyponatremia, hypernatremia, symptomatic hypernatremia, urine osmolarity and vasopressin level	Isotonic saline reduced the incidence of hyponatremia in children who required IVF maintenance therapy
						0.18% saline in 5% dextrose at a standard maintenance rate	56		
						0.18% saline in 5% dextrose at 2/3 of the standard maintenance rate	53		
Kumar 2020 [48]	India	Open-label	Single	12 and 24	Children 3 months to 5 years old who needed IVF for 24 h	0.9% saline in 5% dextrose at a standard maintenance rate	84	Mild hyponatremia, moderate hyponatremia, severe hyponatremia, symptomatic hyponatremia, and hypernatremia	Hypotonic saline did not increase the risk of hyponatremia
						0.45% saline in 5% dextrose at a standard maintenance rate	84		
Lehtiranta 2021 [37]	Finland	Open-label	Single	7 (days)	Acutely ill children aged from 6 months to 12 years and in need of IVF	Plasma-Lyte glucose 50 mg/ml contained 140 mmol/l Na in 5% dextrose, 5 mmol/l of K, 1.5 mmol/l of Mg, 98 mmol/l of Cl, 23 mmol/l of acetate, 23 mmol/l of gluconate at a rate determined by the physician	308	Hypokalemia, hyponatremia, hypernatremia, severe hypokalemia, fluid retention, the proportion of need for fluid therapy change, ICU admission, duration of IVF therapy, hospital stay and the number of deaths	Plasma-Lyte Glucose significantly increased the risk of electrolyte disorders, mainly hypokalemia, compared with hypotonic fluid
						Hypotonic (80 meq/l NaCl, 20 mmol/l Kcl in 5% dextrose at a rate determined by the physician	306		
McNab 2015 [49]	Australia	Double-blinded	Single	At 6 h, then every 24 h until 72 h, or the patient received < 50% of maintenance	Children aged from 3 months to 18 years of age who need maintenance IVF therapy (50–150%)	0.9% saline in 5% dextrose at 80% of the standard maintenance rate	338	Hyponatremia, hypernatremia, severe hyponatremia, severe hypernatremia, hyperchloremia, serum Mg > 1.2 mmol/l, HCO_3_ > 30 mmol/l, weight change, dehydration, overhydration, seizures, and clinically apparent cerebral edema	Isotonic fluid was superior to the hypotonic one in reducing the risk of hyponatremia and did not increase the risk of adverse events
						0.45% saline in 5% dextrose at 80% of the standard maintenance rate	338		
Mierzewska-Schmidt 2015 [36]	Poland	Open-label	Single	2	Children aged 2–12 years who were scheduled for elective ENT surgery	Ringer's acetate	30	Hyponatremia, hypernatremia, hypoglycemia, hyperglycemia, and the change in plasma osmolarity	Isotonic solution (Ringer acetate) was superior to hypotonic fluids in reducing the risk for glucose and electrolyte disturbance in children intraoperatively
						5% glucose in a water solution with 0.9% NaCl with a volume ratio of 2:1	28		
						5% glucose in water solution	33		
Montaana 2008 [50]	Spain	Open-label	Single	6 and 24	Children aged from 29 days to 18 years who required IVF maintenance therapy	Maintenance fluids with a sodium concentration of 140 mEq/L in 5% dextrose	59	Hyponatremia, severe hyponatremia, blood glucose, and blood pressure changes	Isotonic fluid was protective against hospital-acquired hyponatremia without a higher incidence of adverse events
						Maintenance fluids with sodium concentration between 20 and 100 mEq/L in 5% dextrose	63		
Neville 2006 [23]	Australia	Open-label	Single	4	Children aged from 6 months to 14 years with gastroenteritis and in need for IVF therapy	0.9% saline + 2.5% dextrose at a rate determined by the treating physician	51	Mean change of serum Na, serum osmolarity, urine Na, and urine osmolarity in normal saline versus half normal saline group	The isotonic solution was protective against hyponatremia in children with gastroenteritis without causing hypernatremia
						0.45% saline + 2.5% dextrose at a rate determined by the treating physician	51		
Neville 2010 [30]	Australia	Open-label	Single	8 and 24	Children aged 6 months to 15 y with weight > 8 kg who were expected to be taking nothing by mouth for at least 8 h after surgery	0.9% saline + 2.5% dextrose at standard maintenance rate	31	The change in plasma sodium from induction to 8 and 24 h after induction within and between groups, hyponatremia, hypernatremia, changes in ADH level, urinary electrolyte, and osmolarity	Isotonic saline was superior to hypotonic saline in reducing the risk of hyponatremia; however, the fluid restriction was not protective against hyponatremia
						0.9% saline + 5% dextrose at 50% of the standard maintenance rate	31		
						0.45% saline + 2.5% dextrose at standard maintenance rate	31		
						0.45% saline + 5% dextrose at 50% of the standard maintenance rate	31		
Pemde 2014 [51]	India	Blinded	Single	6, 12, 18, and 24	Children aged between 3 mo and 5 y with signs and symptoms suggestive of CNS infection and required intravenous maintenance fluid for at least 24 h	0.9% saline in 5% dextrose at a standard maintenance rate	31	Hyponatremia after 24 h, serum Na values at 0, 6, 12, 18 and 24 h of receiving maintenance fluid	Isotonic intravenous maintenance fluid was superior to hypotonic fluids in reducing the risk of hyponatremia in young children with CNS infections
						0.45% saline in 5% dextrose at a standard maintenance rate	30		
						0.18% saline in 5% dextrose at a standard maintenance rate	31		
Omoifo 2018 [9]	Nigeria	Double-blinded	Single	NR	Children aged between 6 months and 17 years who were scheduled for various minor elective surgical procedures	Normal saline	20	Hyponatremia, serum sodium, and potassium levels	Intraoperative hypotonic fluids are not inferior to isotonic fluids in reducing the risk of hyponatremia in healthy children during minor elective surgeries
						Ringer lactate	20		
						0.18% saline in 4.3% dextrose	25		
Yung 2009 [31]	Australia	Double-blinded	Single	24	Children in the PICU who would normally receive IVF at a traditional maintenance rate for at least 12 h	0.9% saline at 2/3 of the standard maintenance rate	13	Change in serum sodium, change in osmolarity, need for additional fluid boluses, adverse events including seizures, headache, dehydration, and shock	Hypotonic saline at traditional maintenance rates increased the risk of hyponatremia in sick and postoperative children
						0.9% saline at standard maintenance rate	11		
						0.18% saline at 2/3 of the standard maintenance rate	15		
						0.18% saline at standard maintenance rate	11		
Shatabi 2022 [28]	Iran	Double-blinded	Single	2	Infants aged from 1–30 days who required major surgery under general anesthesia	0.9% saline in 5% dextrose	35	Blood glucose levels, serum sodium, serum base excess, pH, bicarbonate levels, potassium levels, heart rate, mean arterial pressure, and oxygen saturation of arterial blood	There was a significant reduction in serum sodium in the hypotonic group
						0.45% saline in 5% dextrose	35		
Torres 2019 [52]	Argentina	Double-blinded	Single	12 and 24	Children aged from 29 days to 15 years with normal serum sodium on admission who required exclusively parenteral maintenance solutions for at least 24 h	0.9% saline in 5% dextrose	155	Hyponatraemia, serum sodium, serum bicarbonate, blood pH, and metabolic acidosis	Hypotonic fluids increased the risk of developing iatrogenic hyponatremia compared to isotonic fluids. The incidence of metabolic acidosis did not differ significantly between the two groups
						0.45% saline in 5% dextrose	163		
Shamim 2014 [53]	India	Open-label	Single	24 and 48	Children aged from 0.5 to 12 years who were admitted and anticipated to receive intravenous fluid for the next 48 h	0.9% saline in 5% dextrose at a rate of 60% of the standard maintenance volume	30	Hyponatremia, serum sodium, serum osmolality, blood urea, serum creatinine, serum potassium, serum chloride, pH, change in weight, urine output, neurological morbidities, and death	Reduced volume isotonic fluid was superior to hypotonic fluid in reducing the risk of hyponatremia in sick children
						0.18% saline in 5% dextrose at a rate of standard maintenance volume	30		
Omoh 2021 [24]	Nigeria	Blinded	Single	8, 16, and 24	Children aged from 2 months to 5 years who required parenteral maintenance fluids for at least 24 h following elective surgery	Ringer lactate	25	Hyponatraemia, normo-natraemia, and changes in mean plasma sodium concentration over the duration of the study	Ringer's lactate was superior to 4.3% dextrose in 0.18% saline in preventing hyponatremia in children aged five years and below during the perioperative period
						0.18% Saline in 4.3% dextrose	25		
Valadao 2015 [22]	Brazil	Double-blinded	Single	24 and 48	Children aged 1–14 years with acute appendicitis that required surgical intervention	0.9% saline and 5% glucose with the volume established by the anesthesiologist	23	Serum sodium, chloride, potassium, and creatinine levels during the intervention and at the end of 48 h, moderate hyponatremia, severe hyponatremia, and hypervolemia were estimated by fluid balance and/or weight gain	The risk of hyponatremia and hypernatremia did not differ significantly between the two groups in the post-appendectomy period
						0.18% saline and 5% glucose with the volume established by the anesthesiologist	27		
Sherazi 2021 [26]	Pakistan	NR	Single	24	Children aged from 1 month to 5 years who were kept NPO and needed maintenance IV fluids	Ringer lactate	78	Serum sodium, hyponatremia, and efficacy in maintaining isonatremia	Isotonic fluids were superior to hypotonic fluids in maintaining normal sodium levels in hospitalized children who required maintenance fluid therapy
						Conventional hypotonic maintenance IV fluid (Plabolyte M)	78		

BET: blood exchange transfusion; STB: serum total bilirubin; SIADH: syndrome of inappropriate secretion of antidiuretic hormone; PMS: parenteral maintenance solutions; PICU: pediatric intensive care unit; CNS: central nervous system; NPO: nothing by mouth; WHO: world health organization

## Efficacy outcomes

### Mild hyponatremia

**Fig. 2:**
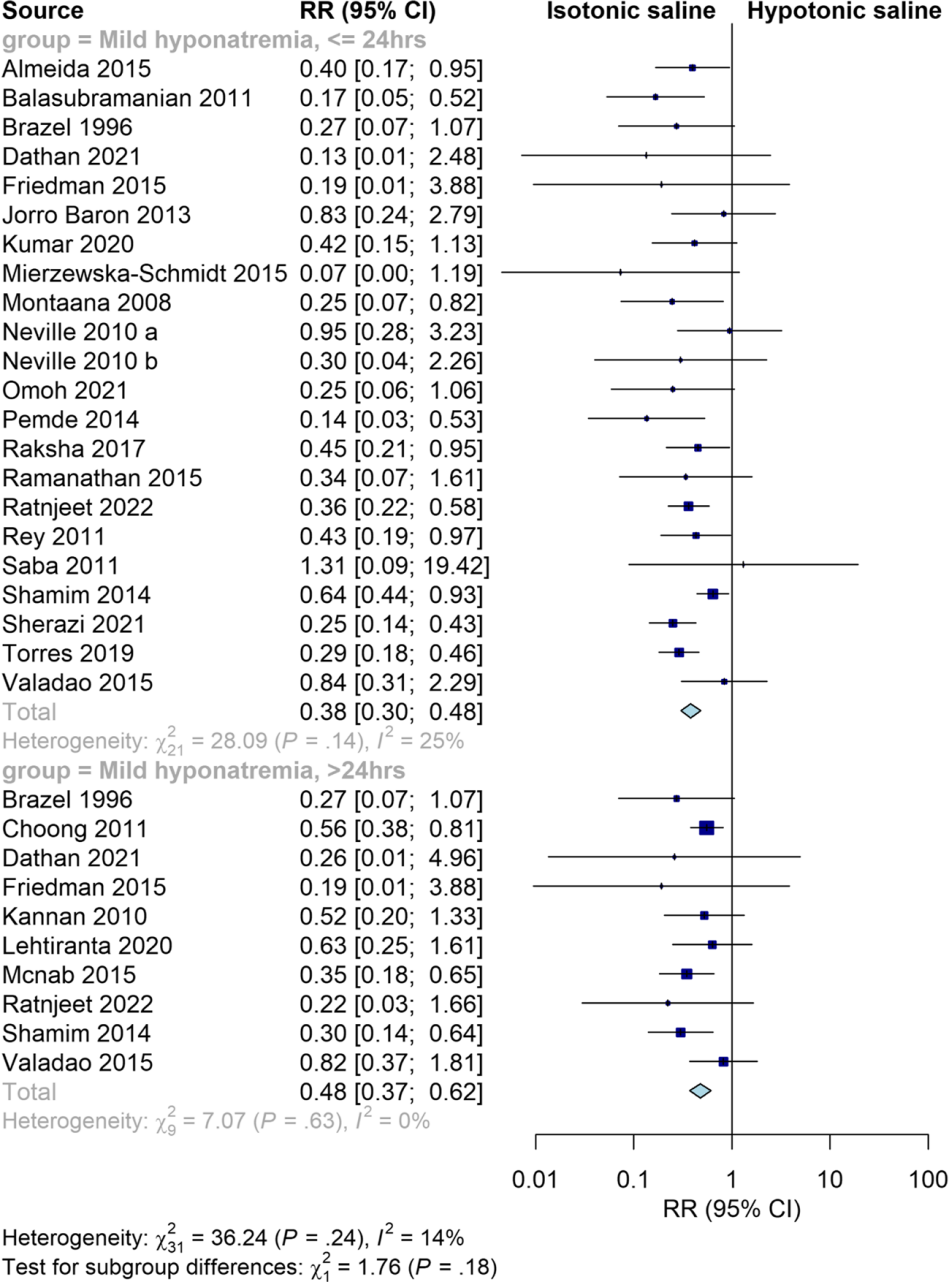
Pooled results for mild hyponatremia at ≤ 24 and > 24 h

Isotonic fluid significantly decreased the risk of mild hyponatremia at both ≤ 24 h and > 24 h (RR = 0.38, 95% CI [0.30, 0.48], *P* < 0.00001; RR = 0.47, 95% CI [0.37, 0.62], *P* < 0.00001, respectively). The pooled results were homogeneous at both points of time (I^2^ = 24%, *P* = 0.15; I^2^ = 0%, *P* = 0.63, respectively) *(*Fig. 2*)*. Our subgroup analysis at 2, 8, 12, 18, 24, 36, 48, and 72 h showed that isotonic fluid decreased the risk of mild hyponatremia at different points of time; the lowest risk was at 2 h followed by 18 h and 36 h (RR = 0.07, 95% CI [0.00, 1.19], *P* = 0.07; RR = 0.21, 95% CI [0.07, 0.59], *P* = 0.003; RR = 0.21, 95% CI [0.07, 0.67], *P* = 0.008, respectively)*.* Our subgroup analysis on isotonic versus moderately hypotonic fluid and isotonic versus severe hypotonic fluid revealed comparable relative risks of mild hyponatremia (RR = 0.39, 95% CI [0.30, 0.50], *P* < 0.00001; RR = 0.37, 95% CI [0.27, 0.51], *P* < 0.00001). This comparable relative risk was also maintained in our subgroup analysis on surgical patients versus medical patients (RR = 0.53, 95% CI [0.36, 0.78], *P* = 0.001; RR = 0.30, 95% CI [0.22, 0.41], *P* < 0.00001, respectively), standard rate versus restricted rate (RR = 0.36, 95% CI [0.26, 0.49], *P* < 0.00001; RR = 0.60, 95% CI [0.34, 1.05], *P* = 0.07, respectively), neonates versus other children (RR = 0.18, 95% CI [0.06, 0.51], *P* = 0.001; RR = 0.39, 95% CI [0.33, 0.46], *P* < 0.00001, respectively), balanced versus 0.9% saline (RR = 0.24, 95% CI [0.15, 0.39], *P* < 0.00001; RR = 0.41, 95% CI [0.34, 0.49], *P* < 0.00001, respectively), and open label studies versus blinded studies (RR = 0.40, 95% CI [0.31, 0.52], *P* < 0.00001; RR = 0.37, 95% CI [0.25, 0.54], *P* < 0.00001, respectively). In addition, our subgroup analysis based on different regions of the included studies showed that isotonic saline significantly decreased the risk of mild hyponatremia in studies conducted in Asia, Australia and Oceania, and Europe (RR = 0.35, 95% CI [0.26, 0.46], *P* < 0.00001; RR = 0.39, 95% CI [0.24, 0.65], *P* = 0.0002; RR = 0.41, 95% CI [0.26, 0.64], *P* = 0.0001, respectively). However, there was no significant difference between the two fluids in terms of mild hyponatremia in studies conducted in both North and South America (RR = 0.39, 95% CI [0.14, 1.10], *P* = 0.07; RR = 0.53, 95% CI [0.23, 1.19], *P* = 0.12, respectively) (Supplementary Fig. 2).

### Moderate hyponatremia

**Fig. 3:**
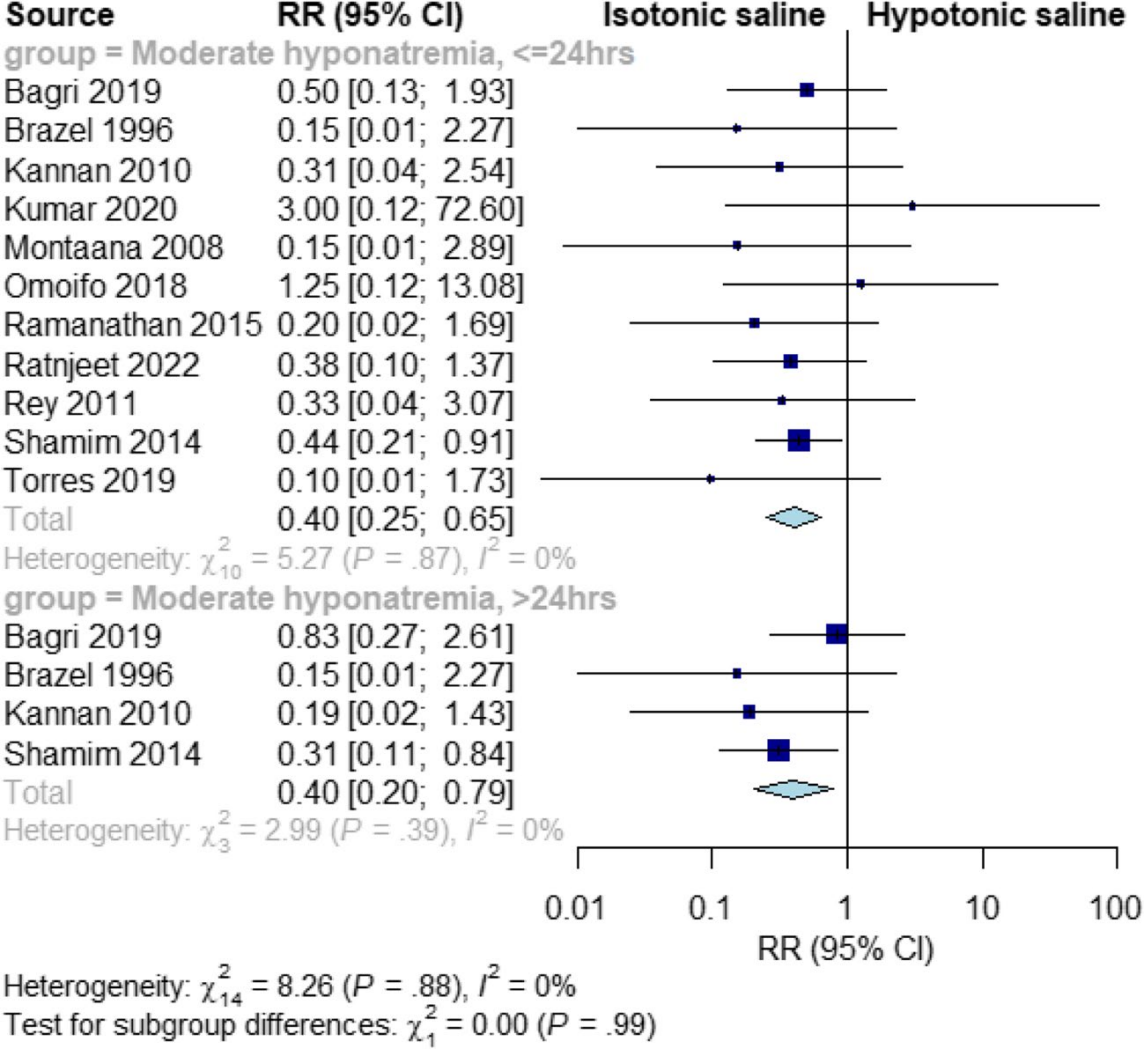
Pooled results for moderate hyponatremia at ≤ 24 and > 24 h

Isotonic fluids significantly decreased the risk of moderate hyponatremia at ≤ 24 and > 24 h, compared to hypotonic fluids (RR = 0.40, 95% CI [0.25, 0.65], *P* = 0.0002; RR = 0.40, 95% CI [0.20, 0.79], *P* = 0.008, respectively). The pooled results were homogeneous at both time points (I^2^ = 0%,* P* = 0.89; I^2^ = 0%, *P* = 0.4, respectively) *(*Fig. 3*).*

Our subgroup analysis at 24, 36, and 72 h showed that isotonic fluid reduced the risk of moderate hyponatremia, with the lowest risk observed at 72 h (RR = 0.17, 95% CI [0.03, 0.88], *P* = 0.03) (Supplementary Fig. 3A).

### Severe hyponatremia

**Fig. 4:**
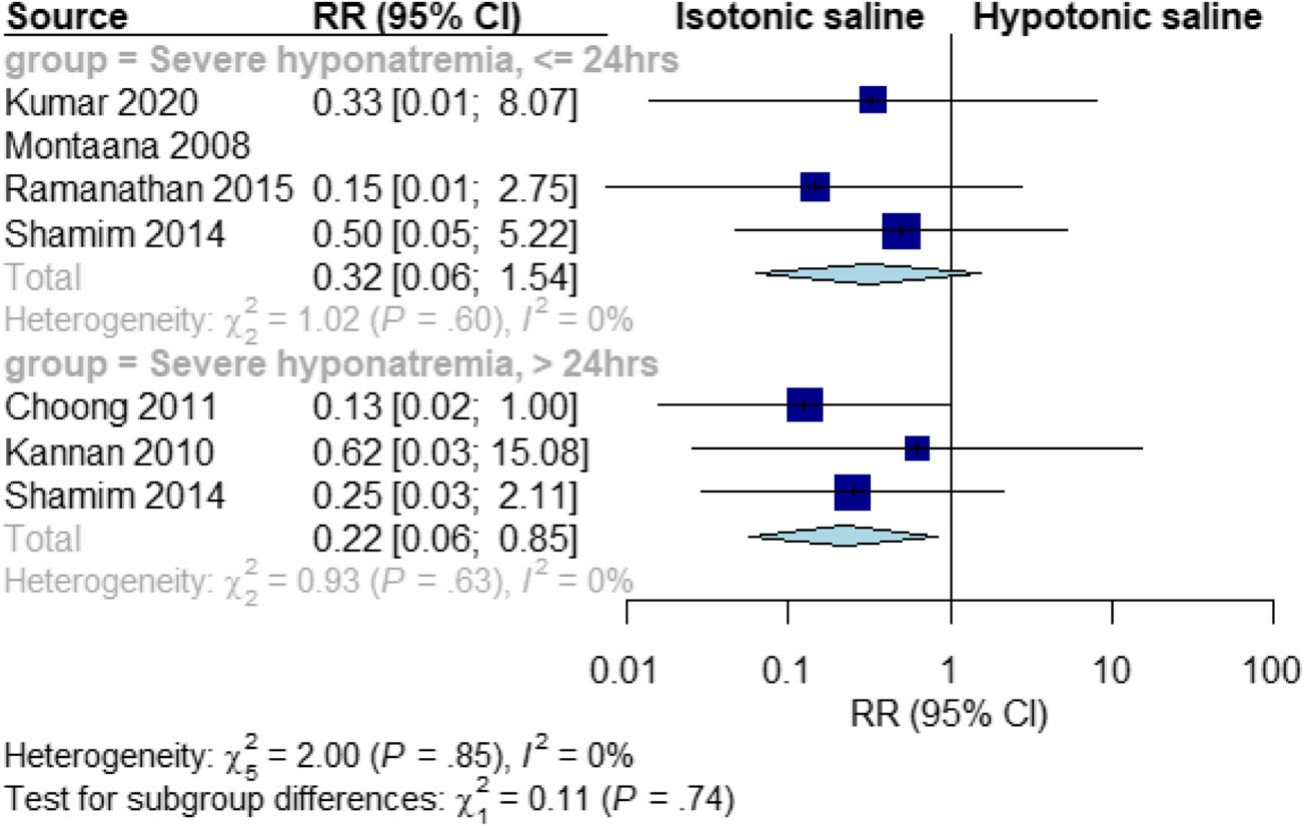
Pooled results for severe hyponatremia at ≤ 24 and > 24 h

Isotonic fluids significantly reduced the risk of severe hyponatremia at > 24 h, compared to hypotonic fluids (RR = 0.22, 95% CI [0.06, 0.85], *P* = 0.03). The pooled results were homogeneous at all-time points (I^2^ = 0%, *P* = 0.7). However, there was no significant difference between the two fluids at ≤ 24 h (RR = 0.32, 95% CI [0.06, 1.54], *P* = 0.15). The pooled results were homogeneous at all time points (I^2^ = 0%, *P* = 0.81) *(*Fig. 4*).*

Our subgroup analysis at 48 h showed that isotonic fluid reduced the risk of severe hyponatremia (RR = 0.18, 95% CI [0.04, 0.78], *P* = 0.02) (Supplementary Fig. 3B).

### Hypernatremia

**Fig. 5:**
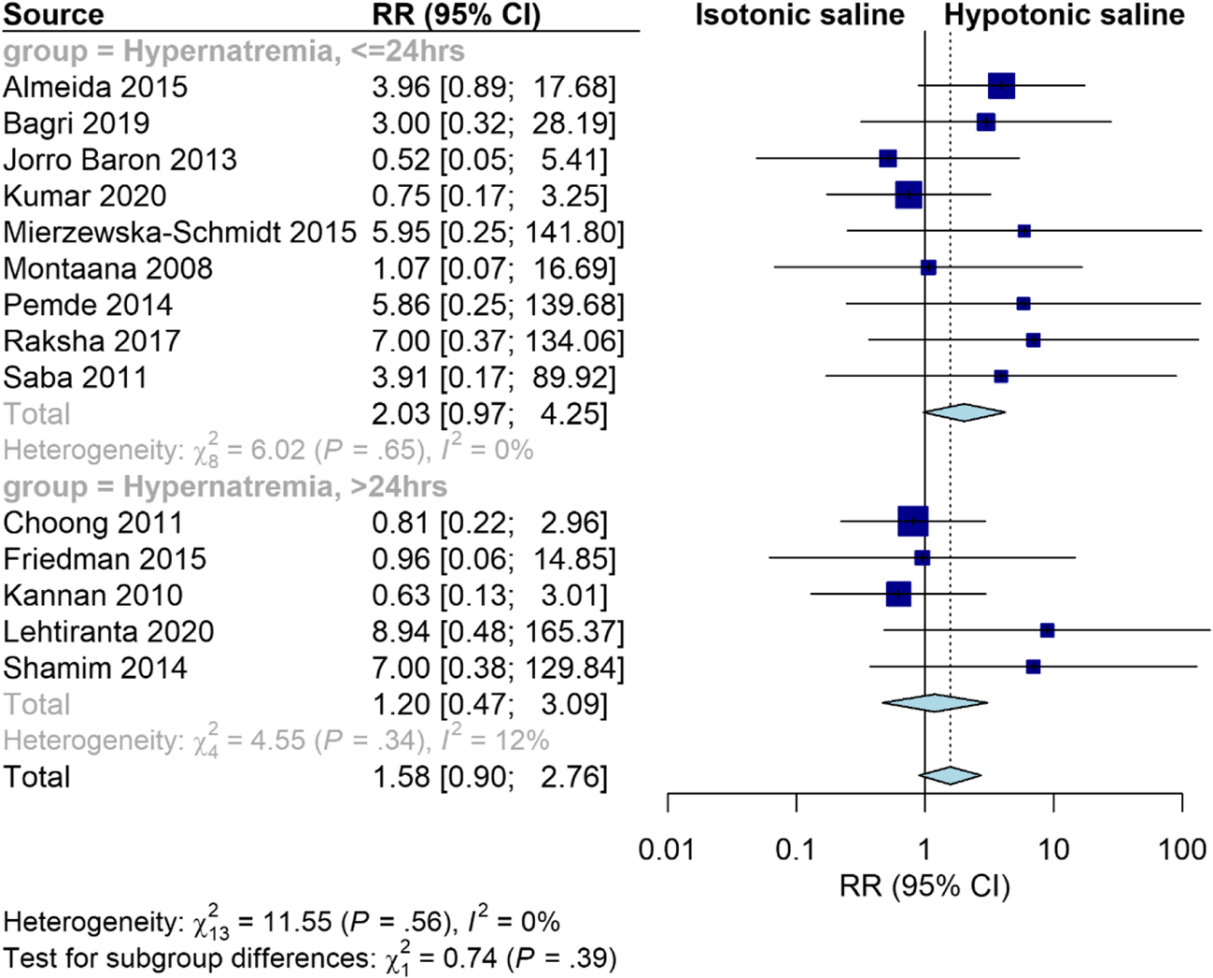
Pooled results for hypernatremia at ≤ 24 and > 24 h. Sensitivity analysis excluding trials conducted on neonates

**Fig. 6:**
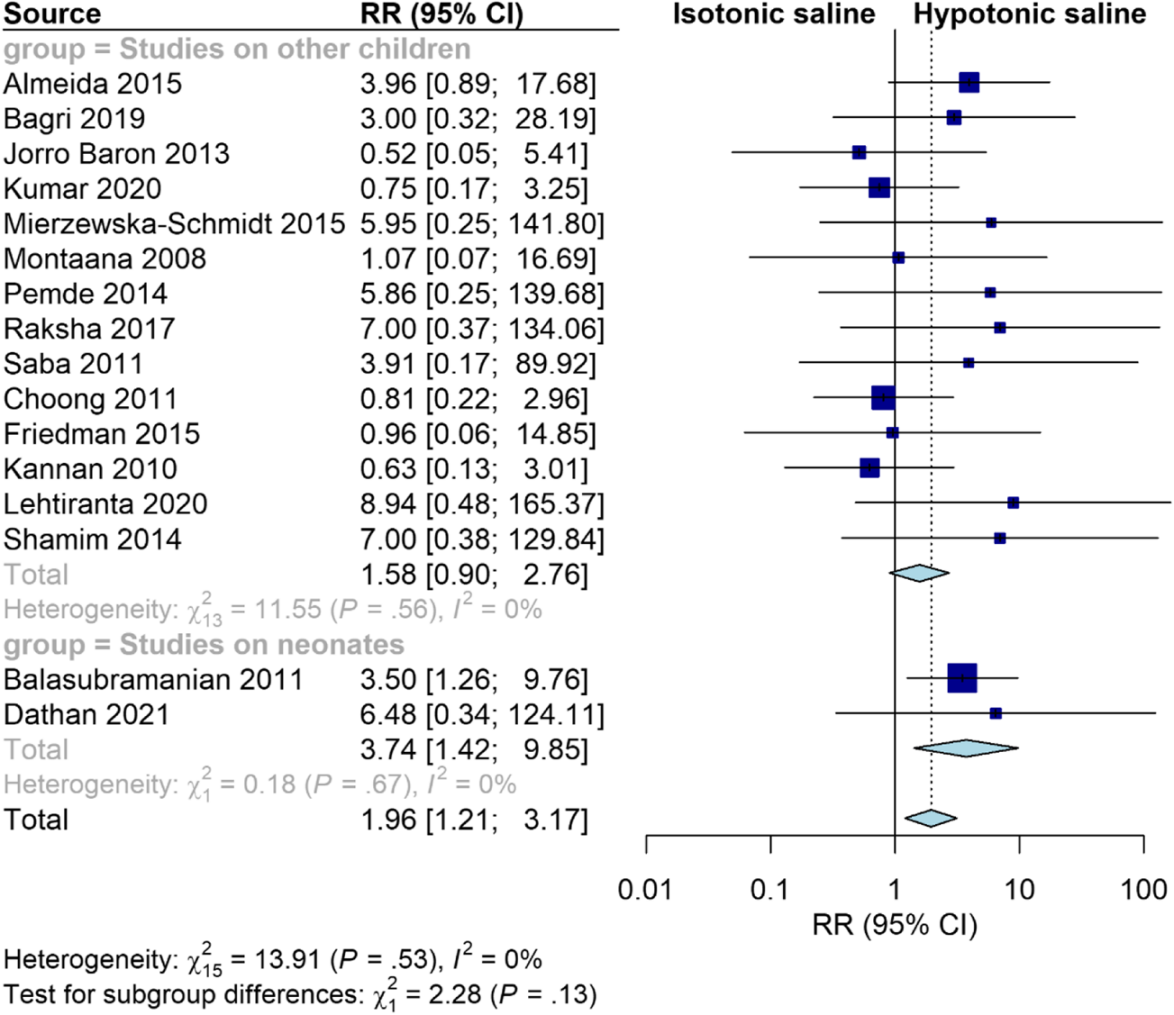
Pooled results for hypernatremia with subgrouping based on the age group

Isotonic fluid significantly increased the risk of hypernatremia at ≤ 24 h compared to hypotonic fluid (RR = 2.44, 95% CI [1.34, 4.45], *P* = 0.003). The pooled results were homogeneous (I^2^ = 0%, *P* = 0.68). However, there was no significant difference between the two groups at > 24 h (RR = 1.42, 95% CI [0.56, 3.59], *P* = 0.47). The pooled results were heterogeneous (I^2^ = 13%, *P* = 0.33) (Supplementary Fig. 4). Interestingly, our sensitivity analysis excluding trials conducted on neonates showed that the risk of hypernatremia became insignificant at both ≤ 24 and > 24 h (RR = 2.03, 95% CI [0.97, 4.25], *P* = 0.06; RR = 1.20, 95% CI [0.47, 3.09], *P* = 0.71, respectively) (Fig. 5). Furthermore, our subgroup analysis based on the age category showed that isotonic fluid significantly increased the risk of hypernatremia in studies conducted on neonates (RR = 3.74, 95% CI [1.42, 9.85], *P* = 0.008), while there was no significant difference between the two fluids in studies conducted on other children (RR = 1.58, 95% CI [0.90, 2.76], *P* = 0.11) *(*Fig. 6*)*.

### Serum sodium

The hypotonic group had significantly lower serum sodium level compared to the isotonic one at ≤ 24 h (MD = –2.36 95% CI [–2.84, –1.88], *P* < 0.00001). The pooled results were heterogeneous (I^2^ = 59%, *P* < 0.0001). However, there was no significant difference between the two groups at > 24 h (MD = –0,92 95% CI [–1.87, 0.03], *P* = 0.06). The pooled results were heterogeneous (I^2^ = 68%, *P* = 0.0009) (Supplementary Fig. 5A).

### Serum osmolarity

The hypotonic group had significantly lower serum osmolarity compared to the isotonic group at ≤ 24 h (MD = –4.85, 95% CI [–6.95, –2.74], *P* < 0.00001). The pooled results were homogeneous (I^2^ = 12%, *P* = 0.33). However, there was no significant difference between the two groups at > 24 h (MD = –8.20, 95% CI [–17.91, 1.52], *P* = 0.10). The pooled results were heterogeneous (I^2^ = 90%, *P* < 0.0001) (Supplementary Fig. 5B).

### Serum chloride

There was a significant difference between the hypotonic and isotonic groups (favoring the hypotonic group) in terms of serum chloride level at ≤ 24 h (MD = –1.68, 95% CI [–2.94, –0.42], *P* = 0.009). The pooled results were heterogeneous (I^2^ = 64%, *P* = 0.004). However, there was no significant difference between the two groups at > 24 h (MD = –0.66, 95% CI [–3.23, 1.90], *P* = 0.61). The pooled results were heterogeneous (I^2^ = 65%, *P* = 0.06) (Supplementary Fig. 5C).

### Serum potassium

There was no significant difference between the isotonic and hypotonic groups in terms of serum potassium level at both the time points, ≤ 24 and > 24 h (MD = 0.00, 95% CI [–0.18, 0.18], *P* = 0.94; MD = 0.01, 95% CI [–0.20, 0.22], *P* = 0.95, respectively). The pooled results were heterogeneous at ≤ 24 and > 24 h (I^2^ = 66%, *P* = 0.001; I^2^ = 74%, *P* = 0.004, respectively) (Supplementary Fig. 5D).

### Serum creatinine

Serum creatinine level was significantly higher in the isotonic group at ≤ 24 h (MD = 0.89, 95% CI [0.84, 0.94], *P* < 0.00001). However, there was no significant difference between the isotonic and hypotonic groups in terms of serum creatinine level at > 24 h (MD = 0.85, 95% CI [–0.02, 1.71], *P* = 0.05). The pooled results were homogeneous  at both ≤ 24 and > 24 h (I^2^ = 0%, *P* = 0.79; I^2^ = 7%, *P* = 0.36) (Supplementary Fig. 5E). Interestingly, our subgroup analysis based on the composition of isotonic fluids revealed that 0.9% saline was associated with significant increase in serum creatinine levels (MD = 0.90, 95% CI [0.84, 0.96], P < 0.00001), while there was no significant difference between isotonic and hypotonic groups in studies which used balanced isotonic solutions (MD = 0.99, 95% CI [–1.91, 3.90], *P* = 0.50) (Supplementary Fig. 5F)*.*

### Blood pH

Isotonic fluid had significantly lower blood pH than hypotonic fluid (MD = –0.05, 95% CI [–0.08 to –0.02], *P* = 0.0006). The pooled results were heterogeneous (I^2^ = 68%, *P* = 0.04) (Supplementary Fig. 6A)*.*

### Blood sugar

There was no significant difference between isotonic and hypotonic fluids in terms of blood sugar (MD = 3.06, 95% CI [–0.45, 6.56], *P* = 0.09). The pooled results were homogeneous (I^2^ = 0%, *P* = 0.78) (Supplementary Fig. 6B).

### Urinary sodium

The hypotonic group had significantly lower urinary sodium than the isotonic group (MD = –37.07, 95% CI [–47.53, –26.61], *P* < 0.00001). The pooled results were homogeneous (I^2^ = 0%, *P* = 0.43) (Supplementary Fig. 6C).

### Length of hospital stay

There was no significant difference between isotonic and hypotonic fluids in terms of length of hospital stay (MD = –0.07, 95% CI [–0.66, 0.51], *P* = 0.8). The pooled results were homogeneous at all time points (I^2^ = 39%, *P* = 0.14) (Supplementary Fig. 6D).

## Safety outcomes

We observed a trend that there was a higher risk for edema and death in the isotonic group compared to the hypotonic group, but this did not reach statistical significance. Therefore, we cannot conclude that one fluid caused more serious harm compared to the other one. Isotonic fluid was comparable to hypotonic one in terms of seizures (RR = 0.47, 95% CI [0.07, 3.15], *P* = 0.38), edema (RR = 1.52, 95% CI [0.88, 2.62], *P* = 0.13), hypertension (RR = 0.92, 95% CI [0.4, 2.13], *P* = 0.85), metabolic acidosis (RR = 1.26, 95% CI [0.84, 1.9], *P* = 0.27), and death (RR = 1.48, 95% CI [0.72, 3.06], *P* = 0.29). The studies were homogeneous (I^2^ = 21%) in seizures and (I^2^ = 0%) in other outcomes (Supplementary Fig. 7).

## Publication bias

The visual representation of the funnel plots along with Egger’s tests depicted non-significant publication bias in assessing the risk ratios of mild hyponatremia, moderate hyponatremia, and hypernatremia (Supplementary Fig. 8).

## GRADE assessment

The GRADE rating results are shown in Supplementary Table 3. According to the GRADE system, the strength of the evidence was high for mild hyponatremia ≤ 24 h and > 24 h, moderate hyponatremia ≤ 24 h and > 24 h, severe hyponatremia > 24 h, and hypernatremia ≤ 24 h; moderate evidence for severe hyponatremia ≤ 24 h and hypernatremia > 24 h.

## Discussion

### Summary of the findings

To the best of our knowledge, this study is the most comprehensive meta-analysis that compares the risk of hyponatremia and hypernatremia following isotonic and hypotonic IV-MFT in hospitalized children. We found that isotonic fluid significantly reduced the risk of mild hyponatremia in hospitalized children. This protective effect of isotonic fluid was maintained when we examined all subgroups except when the outcomes were collected at 2, 6, 16 hours, and at 7 days. In addition, isotonic fluid showed a lower risk of moderate hyponatremia at both ≤ 24 and > 24 h. However, our subgroup analysis at each time point showed that the risk of moderate hyponatremia did not differ at 6, 12, and 48 h. Moreover, isotonic fluid significantly decreased the risk of severe hyponatremia after 24 h but not at ≤ 24 h. This highlights that isotonic fluid is a safer option for longer durations of fluid therapy. In terms of hypernatremia, isotonic fluid increased its risk in neonates only. Regarding the changes in serum electrolytes, we noticed that serum potassium showed no significant difference at both ≤ 24 and > 24 h. Interestingly, hypotonic fluid significantly lowered serum sodium, chloride, and osmolarity, while isotonic fluid significantly increased serum creatinine only at ≤ 24 but not after 24 h. These findings indicate that the mean change in serum sodium, chloride, osmolarity, and creatinine lessens as the fluid is provided for a longer period. Regarding safety outcomes, we found that isotonic fluid was comparable to hypotonic fluids in terms of the risk of seizures, edema, hypertension, and death. Moreover, the length of hospital stay did not differ significantly between the two groups. Together, these findings highlight that isotonic fluid is a safer choice until at least 72 h after IV-MFT.

## Explanation of the findings

Sodium is the main extracellular cation and the main determinant of serum osmolarity. Therefore, its homeostasis is essential to maintain plasma volume. In addition, changes in plasma volume result in dysnatremia [54]. Given that plasma volume is regulated mainly by the antidiuretic hormone (ADH), ADH plays a major role in sodium homeostasis [54, 55]. Increased serum osmolarity is the main stimulus for ADH secretion under normal physiological conditions. However, it is also secreted in response to other non-osmotic stimuli such as dehydration, stress, and pain [55]. These stimuli commonly increase ADH secretion, which further reduces water excretion and triggers dilutional hyponatremia in hospitalized children [42]. Hence, hospitalized children are virtually at risk for developing hyponatremia. Given that hypotonic solutions have a lower concentration of electrolytes compared to that in the plasma, this risk becomes greater with the administration of hypotonic IV-MFT [56]. In contrast, isotonic solutions more closely approximate the plasma sodium concentration [3]. Therefore, they are associated with a lower risk of hyponatremia and lesser changes in serum electrolytes and osmolarity. We believe that the improvement of patients with therapy results in fewer non-osmotic ADH stimuli and, consequently, less water retention and lesser changes in serum osmolarity and electrolytes. Together, this explains the observed significant decrease in serum osmolarity, serum sodium, and serum chloride in the first 24 h of hypotonic IV-MFT and the absence of these changes after 24 h.

In addition to the role of ADH, stress in those pediatric patients induces the activation of the hypothalamic–pituitary–adrenal axis, which stimulates aldosterone secretion, increasing both sodium and water retention [57] (Supplementary Fig. 9). We believe that the observed decrease in urinary sodium concentration in the hypotonic group can be caused by this stress-induced aldosterone secretion. We also suggest that variations in fluid management practice, types of used fluids, or patients’ underlying conditions may explain the differences in hyponatremia following IV-MFT between studies performed in America versus other areas. Overall, further research is required to fully understand this difference. Regarding hypernatremia associated with isotonic IV-MFT, its increased risk in studies conducted on neonates can be attributed to their renal peculiarities, especially if they are premature or affected by any disease.

Despite the observed protective effect of isotonic fluids, one criticism against their use is their associated higher drop in blood pH. This can be attributed to their higher chloride content and lower strong ion difference, which make them more vulnerable to induce hyperchloremic metabolic acidosis (HCMA) [3]. In addition, isotonic IV-MFT is associated with a significant increase in serum creatinine levels in the first 24 h, reflecting the potential risk of a renal concentrating defect, which can also be attributed to the high chloride content in 0.9% saline [58]. This, in turn, leads to renal volume expansion, interstitial fluid retention, and possible renal dysfunction [58]. In contrast, chloride restrictive isotonic solutions, such as the balanced ones, have a lower risk of this potential renal dysfunction, and are therefore better tolerated by the kidneys compared to 0.9% saline [58]. Our subgroup analysis on serum creatinine, based on the composition of isotonic fluids, supports this hypothesis. Given that the volume and rate of IV-MFT are likely to affect kidney function, regardless of fluid type, IV-MFT should be used with caution to avoid this potential risk of renal dysfunction [58].

Although we observed a trend that there was a higher risk of cerebral edema and death in the isotonic group compared to the hypotonic one, a comprehensive evaluation of the causes of death found that they were unrelated to sodium levels or IV-MFT [6, 39, 45–47]. In addition, only a few studies reported severe hyponatremia [42, 46–48, 50, 59] which, in contrast to mild hyponatremia, may be manifested by neurological complications such as cerebral edema and seizures [60]. This may explain why our analysis showed no significant difference between the isotonic and hypotonic fluids in terms of all adverse outcomes. Given the greater susceptibility to develop such complications in pediatric patients [61], we encourage physicians to carefully observe their pediatric patients during IV-MFT until higher quality evidence about these outcomes is developed.

## Agreement and disagreement with previous studies

We summarized our meta-analysis compared with the previous seven meta-analyses that assessed the safety and efficacy of isotonic versus hypotonic fluid in hospitalized children *(*Table 2*)*. Our finding is consistent with all previous meta-analyses concluding that isotonic fluid is safer than hypotonic fluid in terms of hyponatremia. Regarding hypernatremia, only Hasim et al. 2021 showed a significantly increased risk of hypernatremia during the first 24 h of isotonic IV-MFT. In contrast, our results are consistent with the remaining six meta-analyses which show no evidence of this risk. In their analysis, Hasim et al. 2021 included three studies conducted on neonates [25, 28, 40] despite the fact that neonates differ from other children in their renal handling of body fluids and electrolytes [62]. We believe that it was inaccurate to include these studies conducted on neonates with other studies. Therefore, we performed sensitivity analysis excluding these trials. Interestingly, we identified other limitations in the previous meta-analyses. Firstly, despite the variability of used fluids, only two studies performed subgroup analysis based on the duration [14, 15] and rate of IV-MFT [15, 18]. Only three studies performed sub-group analysis based on the concentration of hypotonic fluid [14, 15, 17] and the condition of hospitalized children [15, 18, 20]. This can be attributed to the limited number of clinical trials then. Secondly, we noticed that some studies assessed the absolute values of serum sodium; however, we believe that it is more accurate to assess the change in serum sodium over time. Only our study and Wang et al. 2013 treated each four-arm study (Neville 2010 and Yung 2009) as two separate studies, while other meta-analyses combined the data from groups with different fluid rates together. We believe this may introduce bias. However, we could not avoid this in the included three-arm studies.

**Table 2:** Summary of previous meta-analyses

	Choong 2006 [19]	Wang 2013 [18]	Foster 2014 [17]	McNab 2014 [15]	Yang 2015 [20]	Pauda 2015 [16]	Hasim 2021 [14]	Our study
Number of included studies	6	8	10	10	8	11	22	33
Design of included studies	Clinical trials + observational studies	RCTs	RCTs	RCTs	RCTs	RCTs	RCTs	RCTs
Total sample size	404	855	893	1106	752	1095	3795	5049
Subgrouping based on the duration of fluid interventions	No	No	No	Yes	No	No	Yes	Yes
Subgrouping into surgical versus medical patients	No	Yes	No	Yes	Yes	No	No	Yes
Subgrouping based on the fluid rate	No	Yes	No	Yes	No	No	No	Yes
Subgrouping based on the concentration of hypotonic fluids	No	No	Yes	Yes	No	No	Yes	Yes
Subgrouping based on blinding of personnel in the included studies	No	No	No	No	No	No	No	Yes
Subgrouping based on regions of the included studies	No	No	No	No	No	No	No	Yes
Classified hyponatremia into mild, moderate, and severe	No	Yes	Yes	No	Yes	Yes	No	Yes
Analyzed safety	No	No	No	Yes	No	Yes	Yes	Yes
Hyponatremia	lower with isotonic fluid	lower with isotonic fluid	lower with isotonic fluid	lower with isotonic fluid	lower with isotonic fluid	lower with isotonic fluid	lower with isotonic fluid	lower with isotonic fluid
Hypernatremia	NR	NR	No difference	No difference	No difference	No difference	Lower with hypotonic fluid at ≤ 24 h but no difference at > 24 h	No difference
Serum sodium	Significantly lower with hypotonic fluid	Significantly lower with hypotonic fluid	Significantly lower with hypotonic fluid	Significantly lower with hypotonic fluid	Significantly lower with hypotonic fluid	Significantly lower with hypotonic fluid	Significantly lower with hypotonic fluid at ≤ 24 h but no difference at > 24 h	Significantly lower with hypotonic fluid at ≤ 24 h but no difference at > 24 h
Serum potassium	NR	NR	NR	NR	NR	NR	NR	No difference
Serum osmolarity	NR	NR	NR	NR	NR	NR	Significantly lower with hypotonic fluid at ≤ 24 h but no difference at > 24 h	Significantly lower with hypotonic fluid at ≤ 24 h but no difference at > 24 h
Serum creatinine	NR	NR	NR	NR	NR	NR	NR	Significantly higher with isotonic fluid at ≤ 24 h but no difference at > 24 h
Serum chloride	NR	NR	NR	NR	NR	NR	NR	Significantly lower with hypotonic fluid at ≤ 24 h but no difference at > 24 h
Urinary sodium	NR	NR	NR	Significantly lower with hypotonic fluid	NR	NR	Significantly lower with hypotonic fluid	Significantly lower with hypotonic fluid
Blood sugar	NR	NR	NR	NR	NR	NR	NR	No difference
Blood PH	NR	NR	NR	NR	NR	NR	NR	Significantly lower in the isotonic group
Length of hospital stay	NR	NR	NR	NR	NR	NR	NR	No difference

NR: not reported; RCTs: randomized controlled trials

## Strength points

Our meta-analysis has a larger sample size compared to the previous ones. In addition, it is the first meta-analysis to explore the effect of isotonic versus hypotonic IV-MFT on serum creatinine, serum chloride, serum potassium, blood glucose, blood pH, and the length of hospital stay. Moreover, our study is the first to perform subgroup analysis based on the composition of the isotonic fluid received (i.e., balanced versus 0.9% saline). Furthermore, all the included studies were published RCTs to provide strong evidence. Finally, we evaluated the certainty of the evidence using the GRADE framework.

## Limitations

The main limitation of our study was the heterogeneity of the included studies; a variety of fluids were used at different maintenance rates in children with different medical and surgical conditions. This is why we used the random effect model and performed our subgroup analyses. Despite this heterogeneity, the results were maintained in most subgroup analyses, indicating that we can generalize our findings in a wide range of settings. In addition, due to safety concerns, severe hyponatremia has been reported in only a few studies despite being more associated with adverse effects. Similarly, the small number of reported adverse events may underestimate the true risk of the adverse events associated with IV-MFT. Moreover, six of our included studies [9, 21, 36, 44, 47, 51] were three-arm trials that compared either two types of isotonic or hypotonic fluid with only one type of the other fluid. We combined the data from the two-arm group to compare it with the other group. However, this may introduce bias as the fluids differ in their tonicity or rate of administration. In our surgical versus medical subgroup analysis, we could not include studies with both surgical and medical patients as they did not report data for each group separately. Finally, a significant number of patients likely received a fluid bolus, which could presumably be isotonic, prior to continuing maintenance fluids, and this could impact serum sodium concentration along with other variables.

## Implications of these findings in practice

The results of our study indicate that isotonic IV-MFT reduces the risk of iatrogenic hyponatremia. These results apply for the first 72 h with different administration rates in both medical and surgical pediatric patients. However, because hyponatremia can occur with both isotonic and hypotonic IV-MFT, physicians should consider the special needs of each child when prescribing IV-MFT, together with crucial monitoring of kidney function. Given the greater susceptibility of pediatric patients to develop neurological complications, such as cerebral edema and seizures [61], we encourage physicians to carefully observe their pediatric patients during IV-MFT until higher quality evidence about these complications is developed. Finally, we suggest the use of balanced isotonic solutions rather than high-chloride fluids (i.e., 0.9% saline) to avoid the potential risk of renal dysfunction associated with isotonic IV-MFT, especially in neonates.

## Recommendations

Regarding future research, we recommend doing trials for > 72 h to investigate the true effect of IV-MFT on sodium and electrolyte balance when it is administered for a longer duration. Only one study reported no significant difference between isotonic and hypotonic fluid after 7 days of IV-MFT [37]. Therefore, there is a lack of knowledge about the actual effect of IV-MFT when it is administered for a longer duration. We also recommend including ADH and HCMA as outcomes of interest because they were reported in only a few studies. Therefore, we could not investigate the effect of isotonic IV-MFT on the development of HCMA. We recommend future studies to validate this point.

## Conclusion

Isotonic IV-MFT was superior to hypotonic fluids in reducing the risk of iatrogenic hyponatremia. However, it significantly increased the risk of hypernatremia in neonates. Another criticism against the use of isotonic fluids is the potential risk of renal dysfunction, which is reflected by the significant increase of serum creatinine and decrease of blood pH. Given that the risk of hypernatremia is not important even in neonates and the kidney tolerates chloride restrictive isotonic solutions, such as balanced ones, better than 0.9% saline, we propose to use balanced isotonic IV-MFT in hospitalized children.

### Supplementary Information

Below is the link to the electronic supplementary material.

Graphical abstract (PPTX 1452 KB)

Supplementary file1 (DOCX 32 KB)

Supplementary file2 (DOCX 1455 KB)

Supplementary file3 (DOCX 9735 KB)

Supplementary file4 (DOCX 3077 KB)

Supplementary file5 (DOCX 577 KB)

Supplementary file6 (DOCX 14408 KB)

Supplementary file7 (DOCX 5009 KB)

Supplementary file8 (DOCX 54 KB)

Supplementary file9 (DOCX 4596 KB)

Supplementary file10 (DOCX 2558 KB)

Supplementary file11 (DOCX 18 KB)

Supplementary file12 (DOCX 88 KB)

Supplementary file13 (DOCX 28 KB)

## Data Availability

The datasets used and/or analyzed during the current study are available from the corresponding author upon reasonable request.
