# Supplementary material for: Efficacy and safety of isotonic versus hypotonic intravenous maintenance fluids in hospitalized children: an updated systematic review and meta-analysis of randomized controlled trials
Source: Pediatr Nephrol. 2023 Jun 26;39(1):57–84. doi: 10.1007/s00467-023-06032-7 (PMC10673968; doi:10.1007/s00467-023-06032-7)
Supplement: Supplementary file 1 — Graphical abstract (PPTX 1452 KB) [file 467_2023_6032_MOESM1_ESM.pptx]

## Slide 1
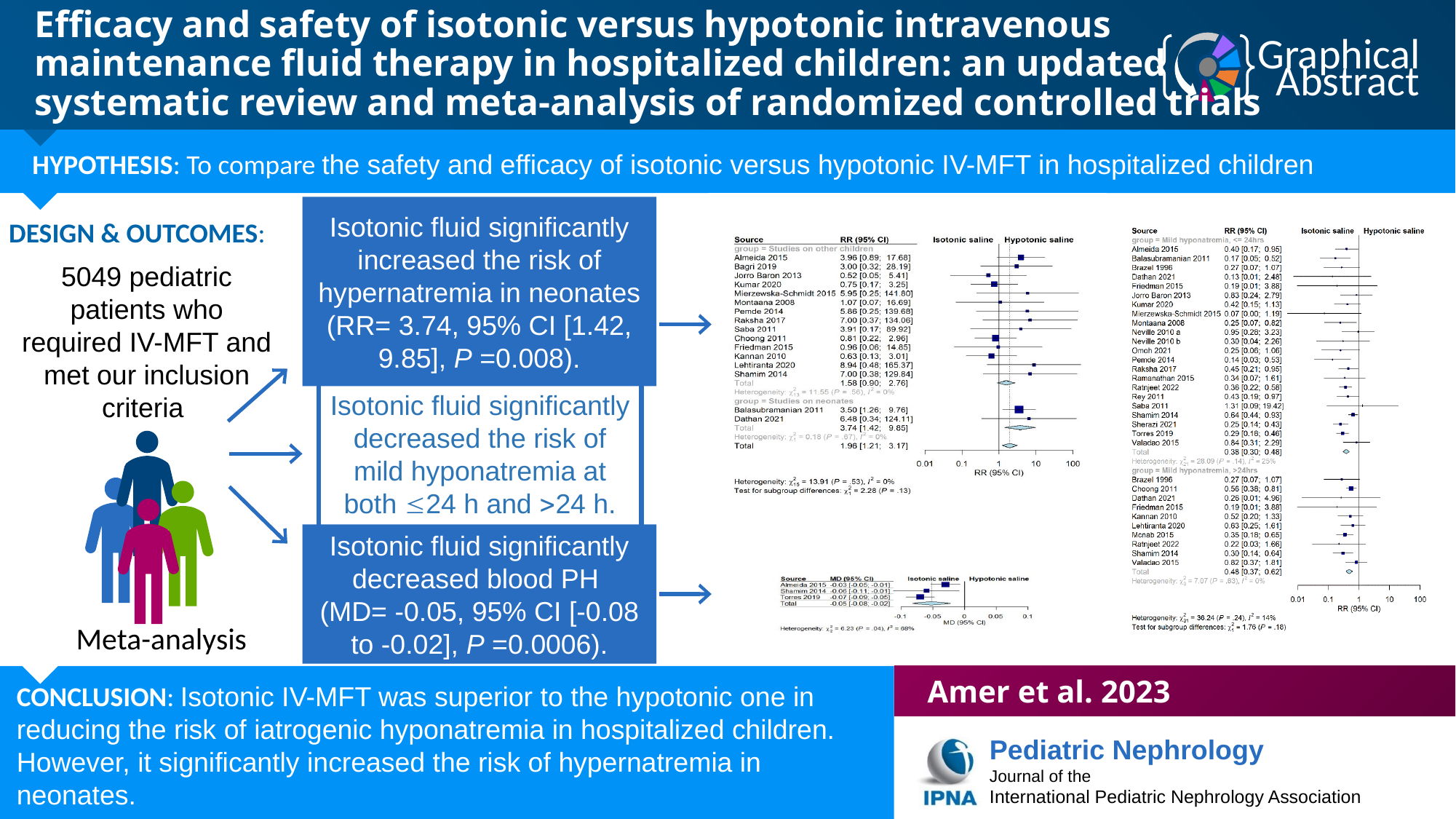

Efficacy and safety of isotonic versus hypotonic intravenous
maintenance fluid therapy in hospitalized children: an updated
systematic review and meta-analysis of randomized controlled trials
HYPOTHESIS: To compare the safety and efficacy of isotonic versus hypotonic IV-MFT in hospitalized children
Isotonic fluid significantly increased the risk of hypernatremia in neonates (RR= 3.74, 95% CI [1.42, 9.85], P =0.008).
DESIGN & OUTCOMES:
5049 pediatric patients who required IV-MFT and met our inclusion criteria
Isotonic fluid significantly decreased the risk of mild hyponatremia at both ≤24 h and 24 h.
Isotonic fluid significantly decreased blood PH
(MD= -0.05, 95% CI [-0.08 to -0.02], P =0.0006).
Meta-analysis
Amer et al. 2023
CONCLUSION: Isotonic IV-MFT was superior to the hypotonic one in reducing the risk of iatrogenic hyponatremia in hospitalized children. However, it significantly increased the risk of hypernatremia in neonates.
