# Supplementary material for: Efficacy and safety of isotonic versus hypotonic intravenous maintenance fluids in hospitalized children: an updated systematic review and meta-analysis of randomized controlled trials
Source: Pediatr Nephrol. 2023 Jun 26;39(1):57–84. doi: 10.1007/s00467-023-06032-7 (PMC10673968; doi:10.1007/s00467-023-06032-7)
Supplement: Supplementary file 3 — Supplementary file2 (DOCX 1455 KB) [file 467_2023_6032_MOESM3_ESM.docx]

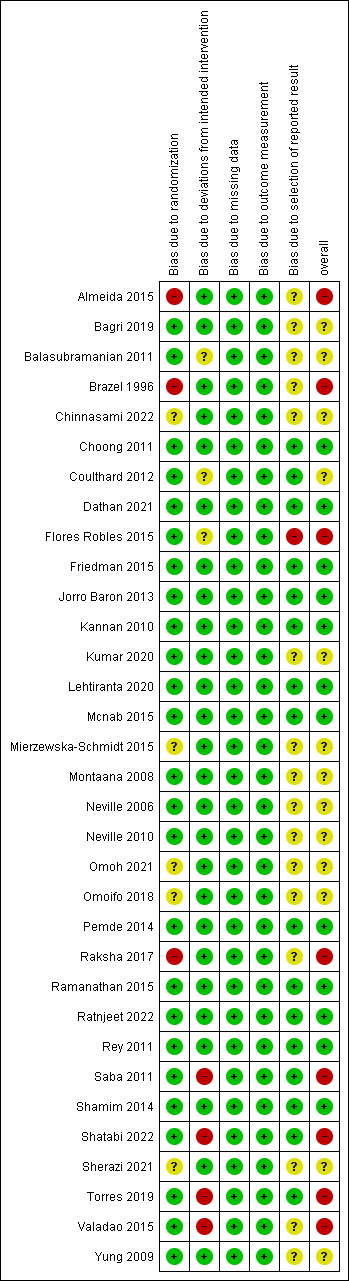


**Supplementary Fig. 1A** Risk of bias summary of the included studies





**Supplementary Fig. 1B** Risk of bias graph of the included studies
