# Supplementary material for: Efficacy and safety of isotonic versus hypotonic intravenous maintenance fluids in hospitalized children: an updated systematic review and meta-analysis of randomized controlled trials
Source: Pediatr Nephrol. 2023 Jun 26;39(1):57–84. doi: 10.1007/s00467-023-06032-7 (PMC10673968; doi:10.1007/s00467-023-06032-7)
Supplement: Supplementary file 4 — Supplementary file3 (DOCX 9735 KB) [file 467_2023_6032_MOESM4_ESM.docx]

**A**

**

**

**B**

**
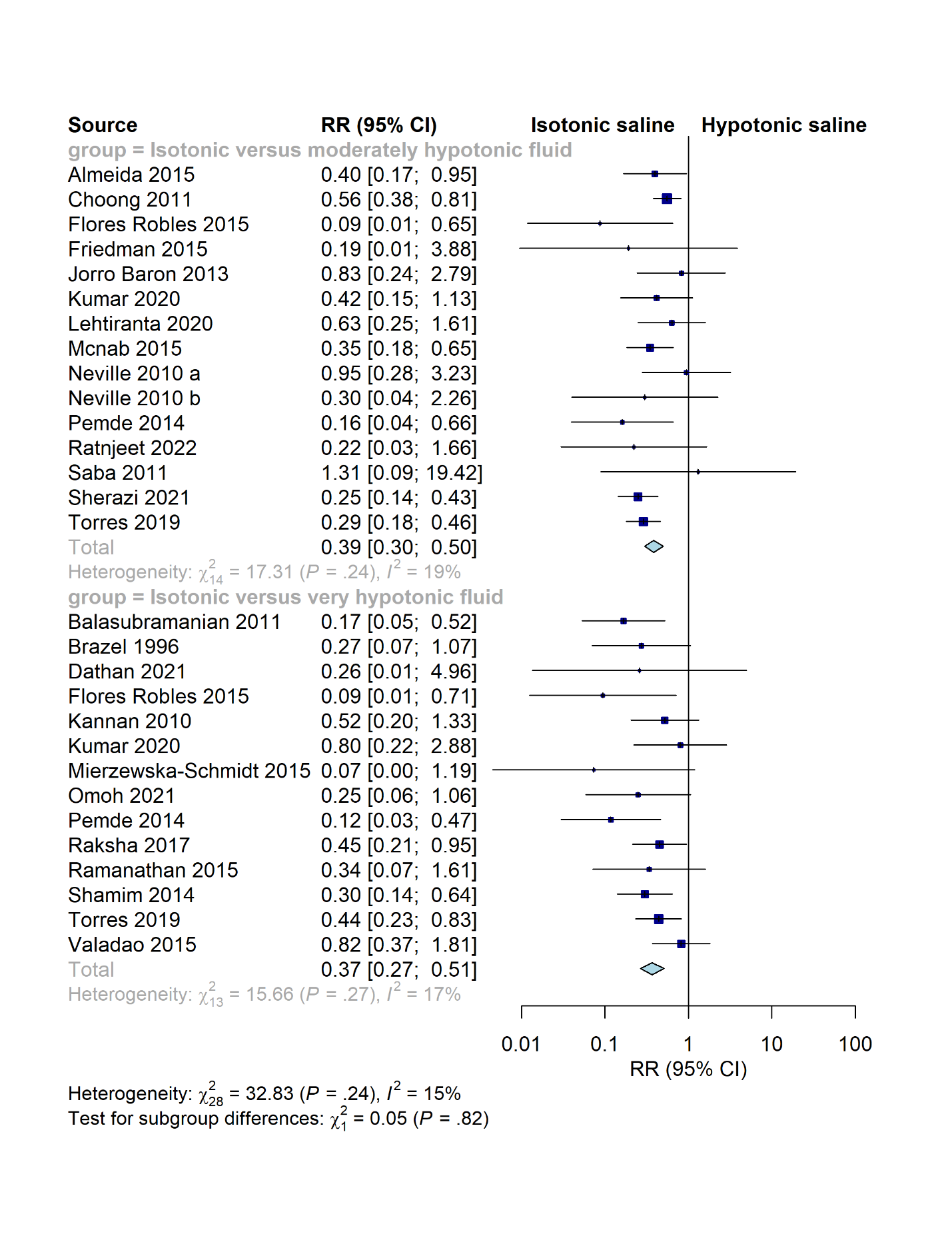
**

**C**

**
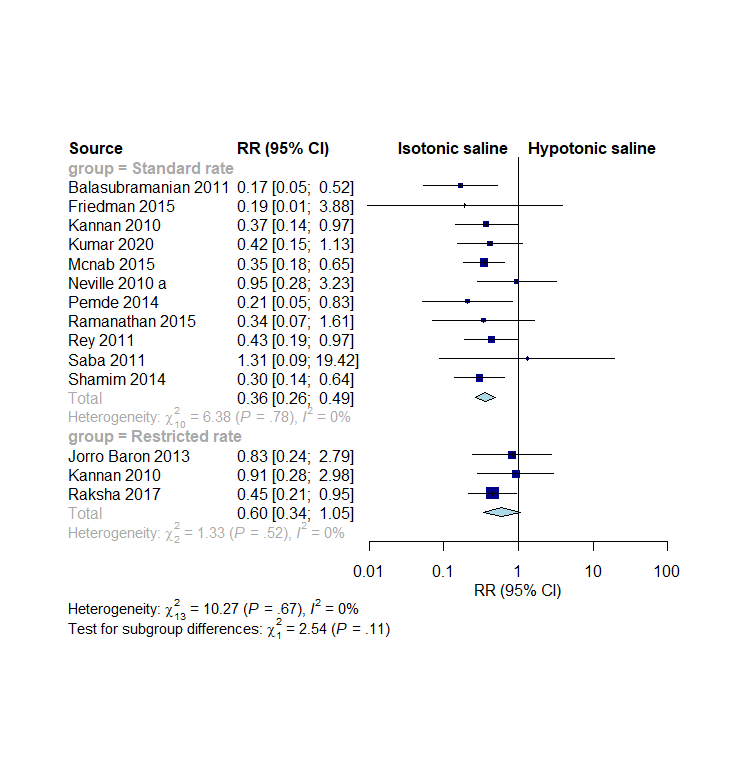
**

**D**

**
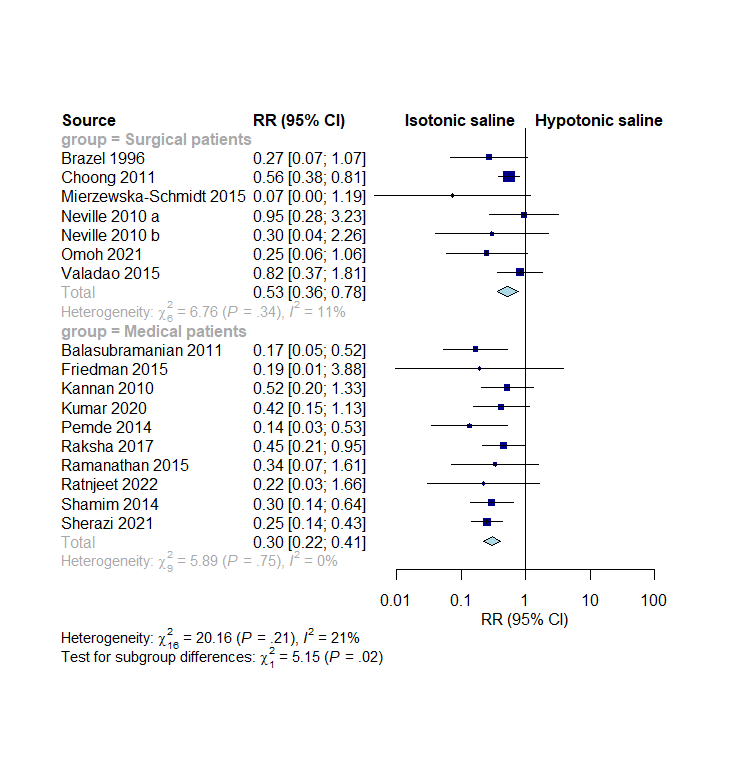
**

**E
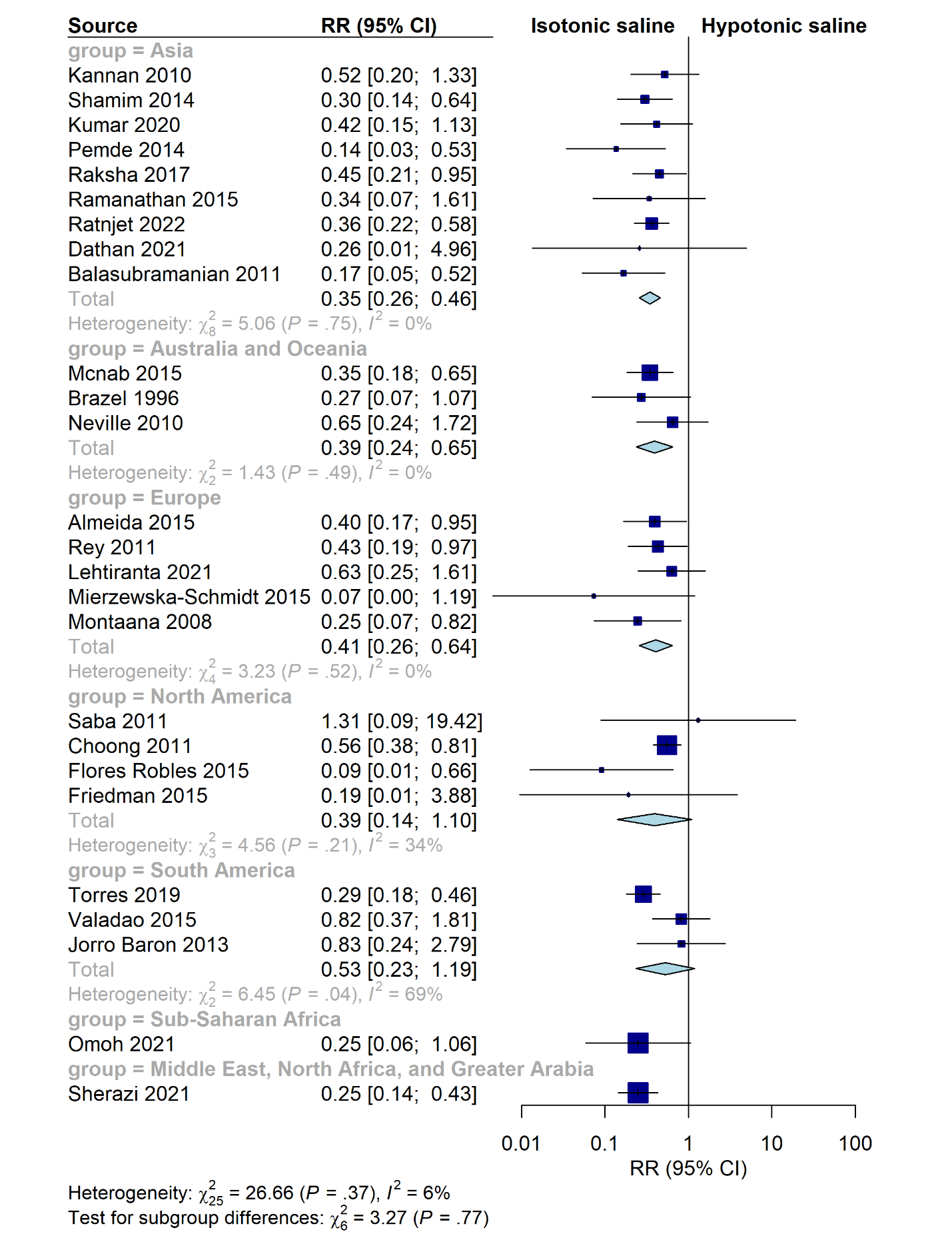
**

**F**

**
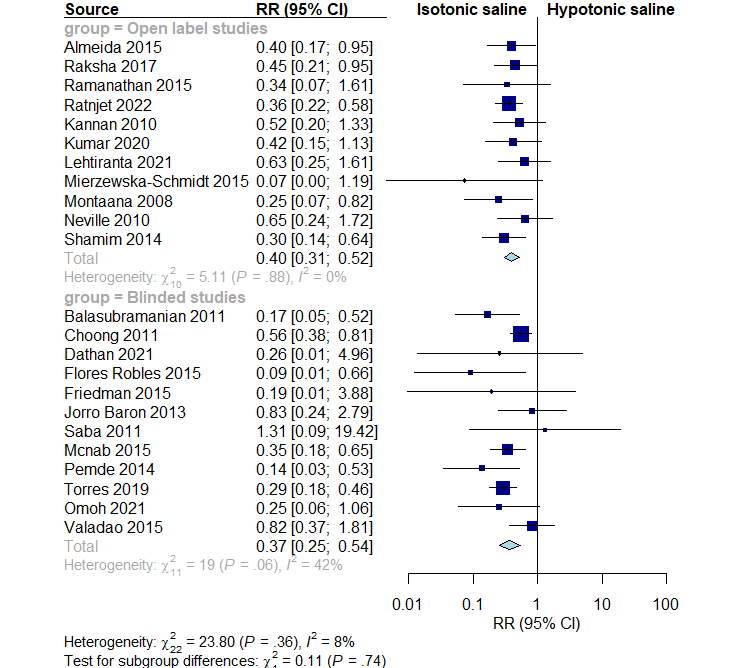
**

**G**

**
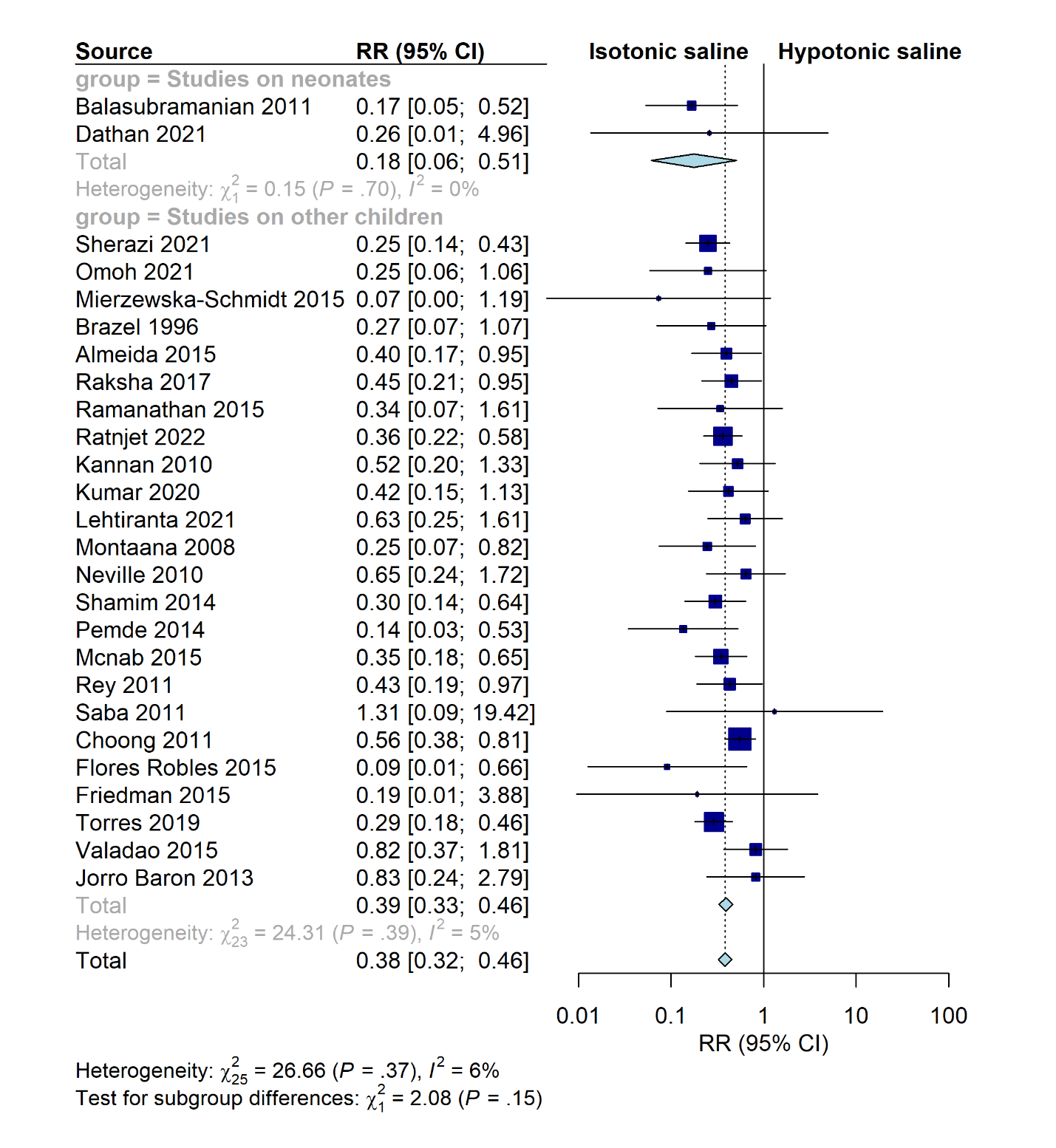
**

**H**

**
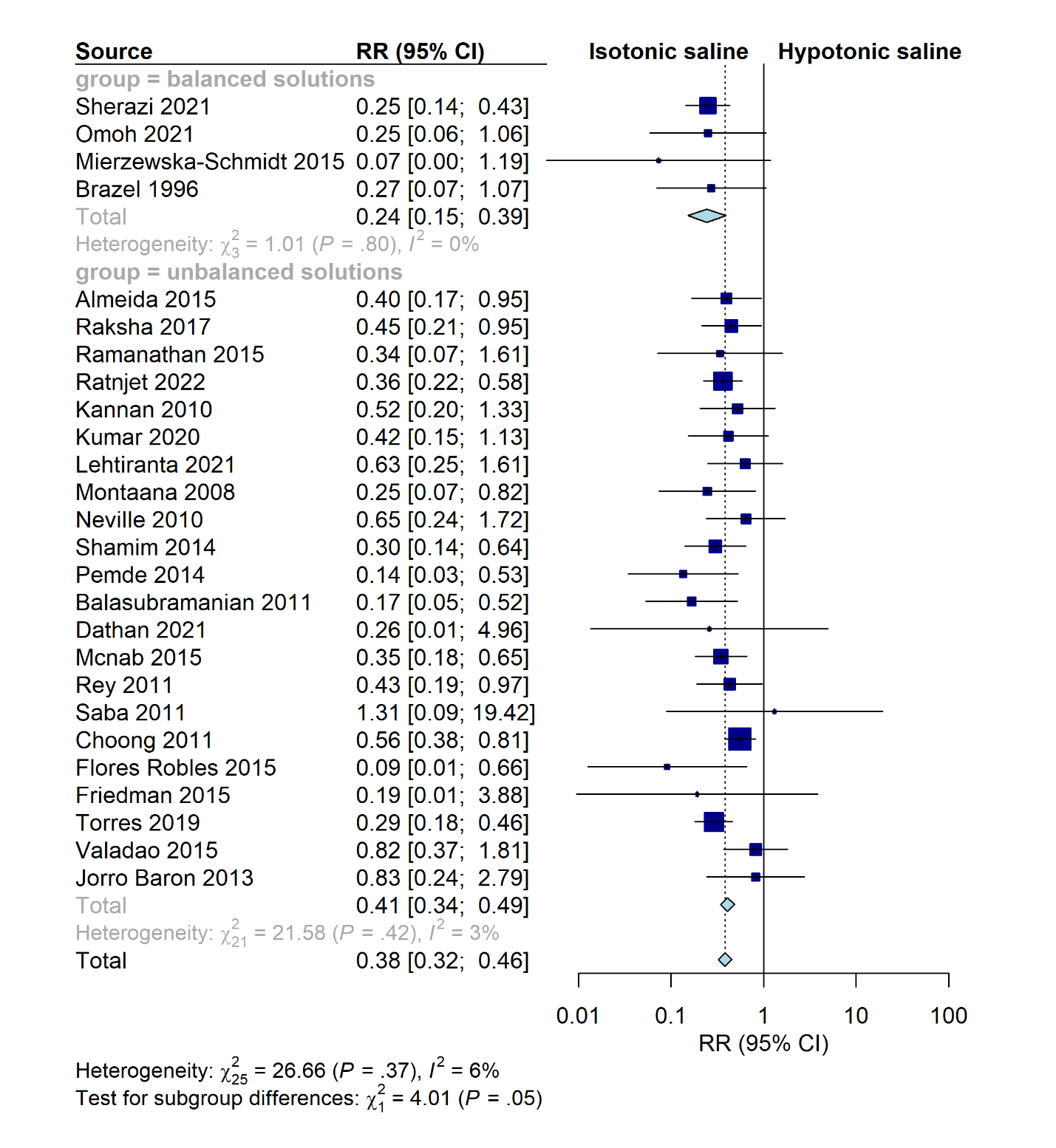
**

**Supplementary Fig. 2** Forest plots showing the risk of developing mild hyponatremia in hospitalized children following isotonic and hypotonic fluids with subgrouping based on A) different time points, B) concentration of hypotonic fluid, C) hypotonic fluid rate, D) condition of hospitalized children, E) regions of included studies, F) blinding of study personnel, G) age category, and H) composition of isotonic fluids
