# Supplementary material for: Efficacy and safety of isotonic versus hypotonic intravenous maintenance fluids in hospitalized children: an updated systematic review and meta-analysis of randomized controlled trials
Source: Pediatr Nephrol. 2023 Jun 26;39(1):57–84. doi: 10.1007/s00467-023-06032-7 (PMC10673968; doi:10.1007/s00467-023-06032-7)
Supplement: Supplementary file 5 — Supplementary file4 (DOCX 3077 KB) [file 467_2023_6032_MOESM5_ESM.docx]

**A**

**
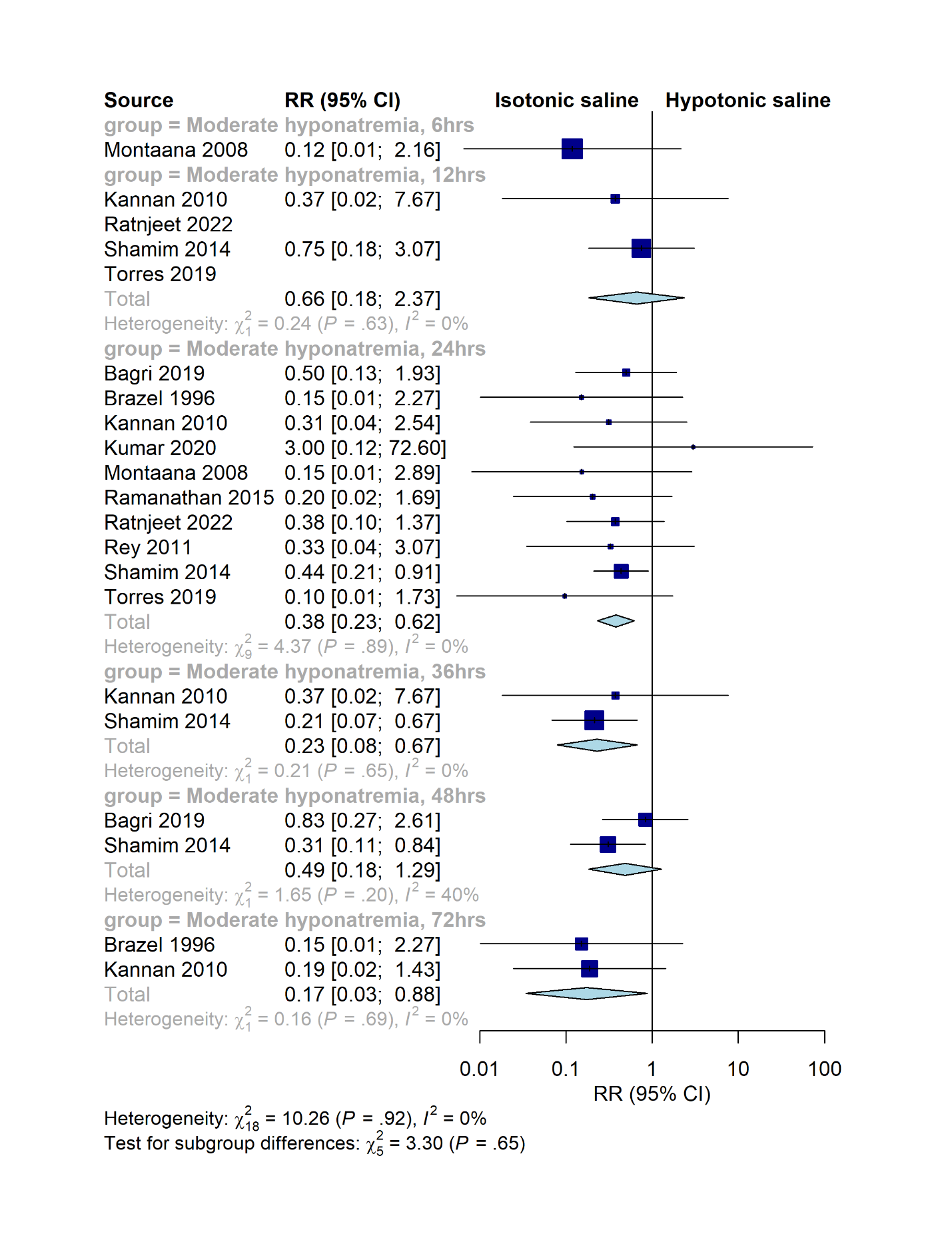
**

**B**

**
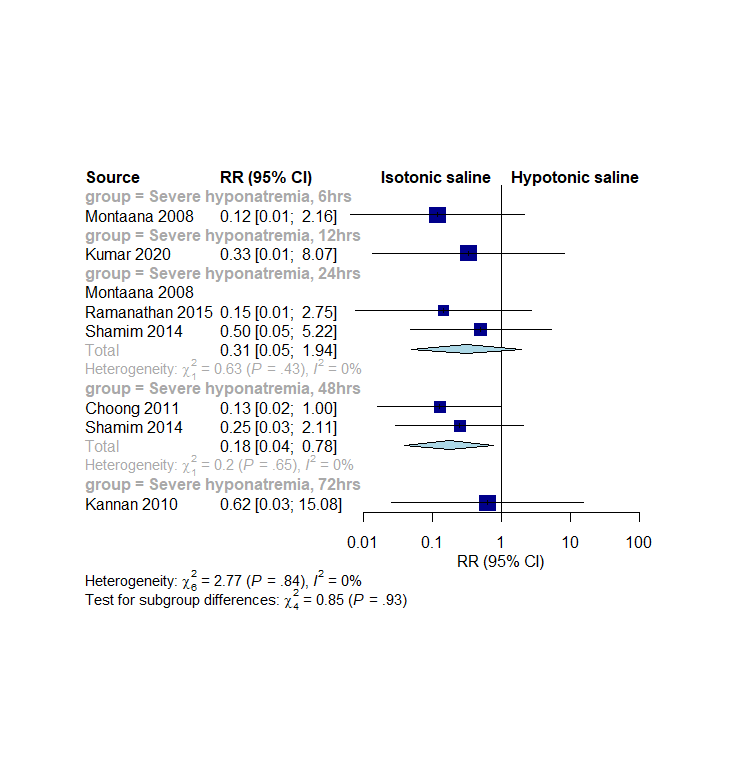
**

**Supplementary Fig. 3** Forest plots showing the risk of developing A) moderate hyponatremia and B) severe hyponatremia at different time points following isotonic versus hypotonic fluids in hospitalized children
