# Supplementary material for: Efficacy and safety of isotonic versus hypotonic intravenous maintenance fluids in hospitalized children: an updated systematic review and meta-analysis of randomized controlled trials
Source: Pediatr Nephrol. 2023 Jun 26;39(1):57–84. doi: 10.1007/s00467-023-06032-7 (PMC10673968; doi:10.1007/s00467-023-06032-7)
Supplement: Supplementary file 6 — Supplementary file5 (DOCX 577 KB) [file 467_2023_6032_MOESM6_ESM.docx]

**Supplementary Fig. 4** Pooled results for hypernatremia at ≤24 and >24 before excluding studies conducted on neonates
