# Supplementary material for: Efficacy and safety of isotonic versus hypotonic intravenous maintenance fluids in hospitalized children: an updated systematic review and meta-analysis of randomized controlled trials
Source: Pediatr Nephrol. 2023 Jun 26;39(1):57–84. doi: 10.1007/s00467-023-06032-7 (PMC10673968; doi:10.1007/s00467-023-06032-7)
Supplement: Supplementary file 7 — Supplementary file6 (DOCX 14408 KB) [file 467_2023_6032_MOESM7_ESM.docx]

**A**

**
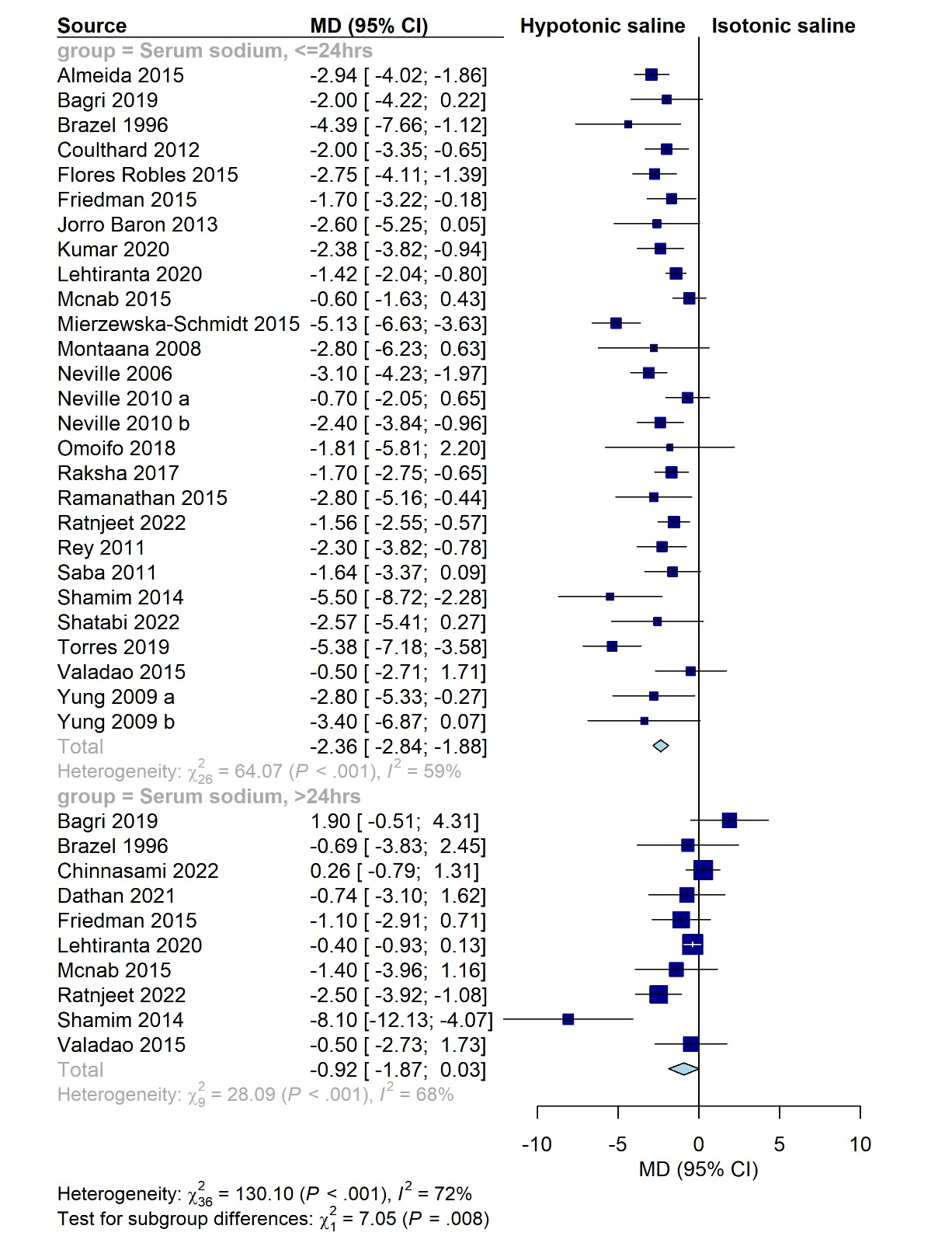
**

**B**

**
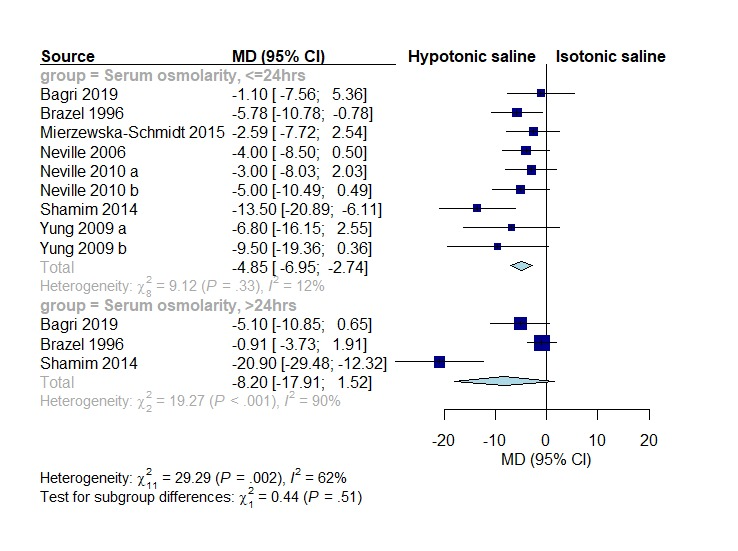
**

**C**

**
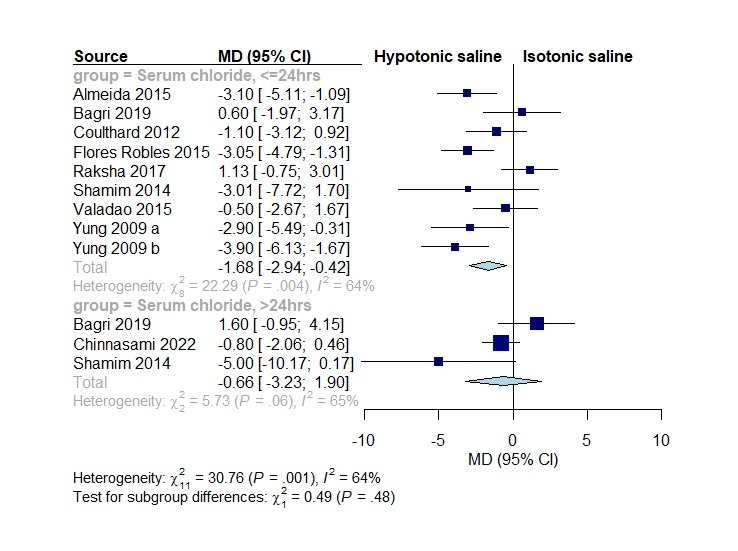
**

**D**

**
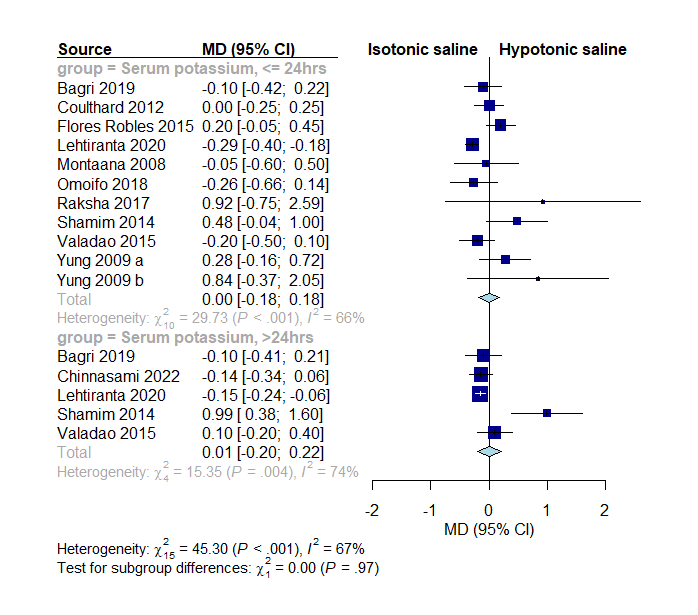
**

**E**

**
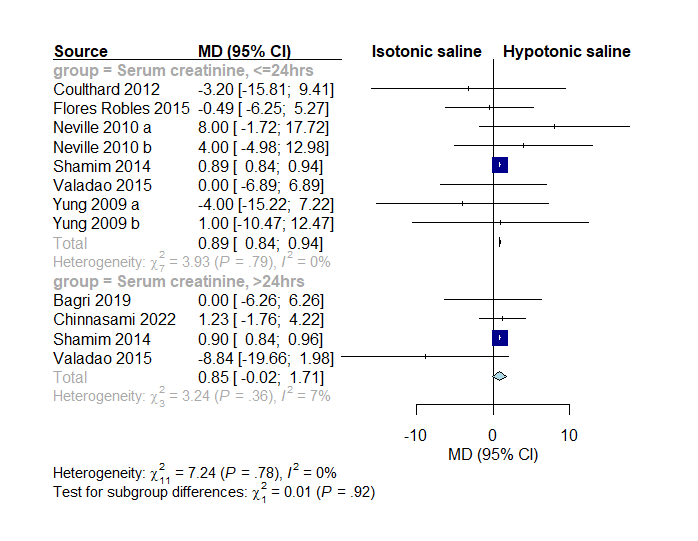
**

**Supplementary Fig 5** Forest plots showing the change in A) serum sodium, B) serum osmolarity, C) serum chloride, D) serum potassium, and E) serum creatinine following isotonic and hypotonic fluids in hospitalized children at ≤ 24 hours and > 24 hours

**
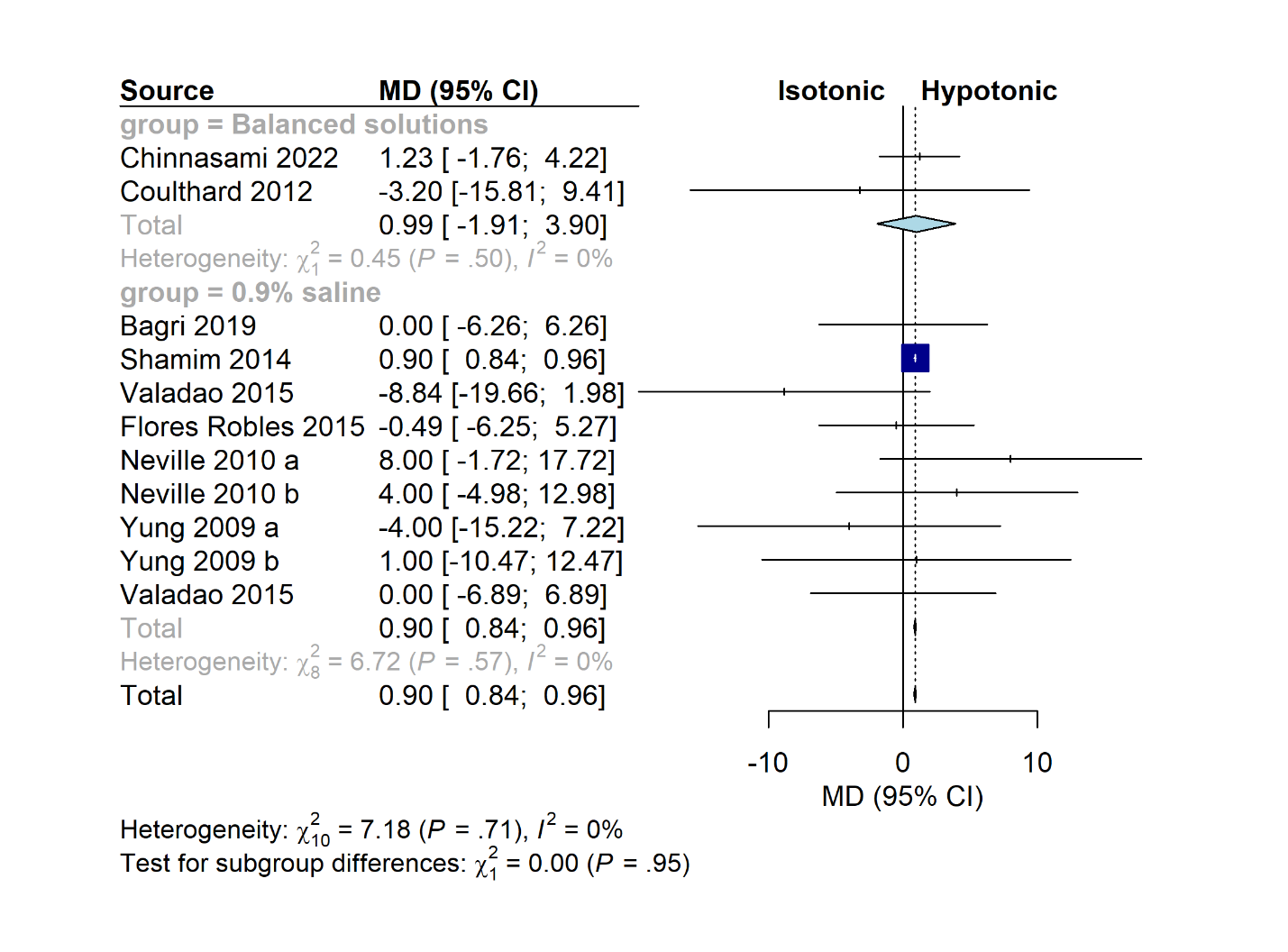
**

**Supplementary Fig. 5F** pooled results for serum creatinine with subgrouping based on the composition of isotonic fluids
