# Supplementary material for: Efficacy and safety of isotonic versus hypotonic intravenous maintenance fluids in hospitalized children: an updated systematic review and meta-analysis of randomized controlled trials
Source: Pediatr Nephrol. 2023 Jun 26;39(1):57–84. doi: 10.1007/s00467-023-06032-7 (PMC10673968; doi:10.1007/s00467-023-06032-7)
Supplement: Supplementary file 8 — Supplementary file7 (DOCX 5009 KB) [file 467_2023_6032_MOESM8_ESM.docx]

**A**

**
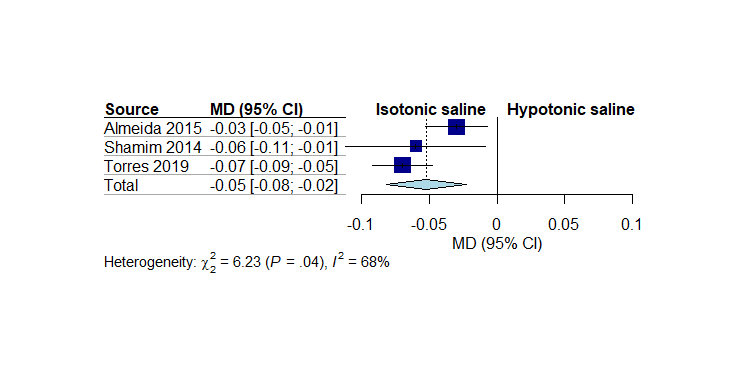
**

**B
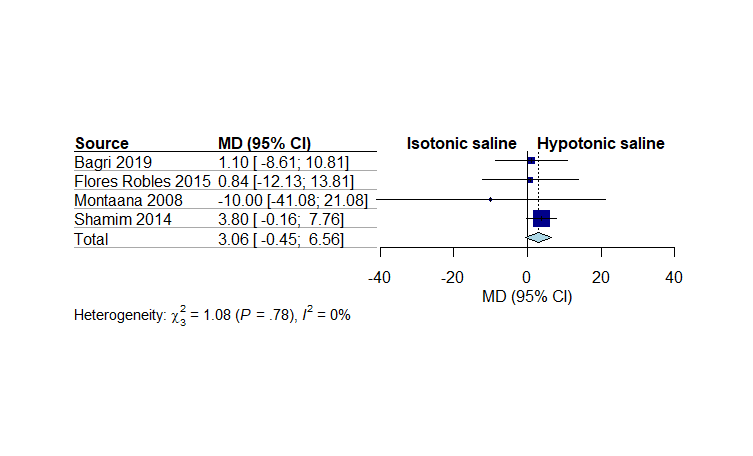
**

**C**

**
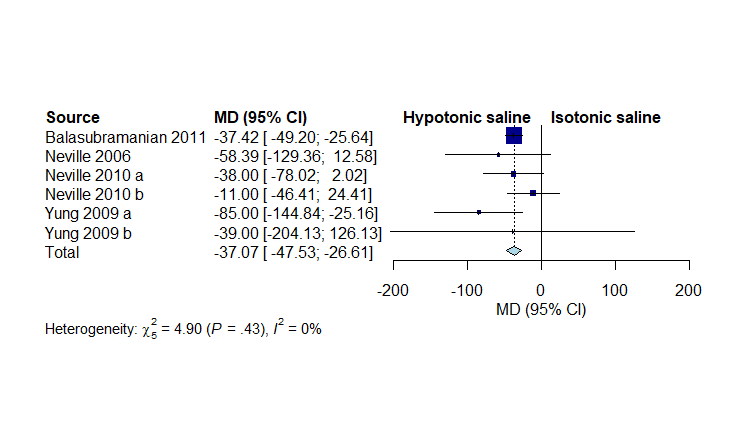
**

**D**

**
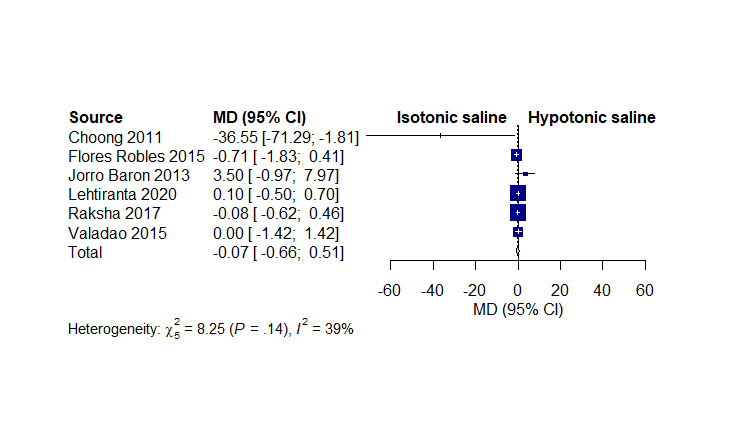
**

**Supplementary Fig. 6** Forest plots showing A) blood PH, B) blood sugar, C) urine sodium, and D) the length of hospital stay following isotonic and hypotonic fluids in hospitalized children
