# Supplementary material for: Efficacy and safety of isotonic versus hypotonic intravenous maintenance fluids in hospitalized children: an updated systematic review and meta-analysis of randomized controlled trials
Source: Pediatr Nephrol. 2023 Jun 26;39(1):57–84. doi: 10.1007/s00467-023-06032-7 (PMC10673968; doi:10.1007/s00467-023-06032-7)
Supplement: Supplementary file 9 — Supplementary file8 (DOCX 54 KB) [file 467_2023_6032_MOESM9_ESM.docx]

1. **Death**

**

**

1. **Seizures**

**

**

1. **Hypertension**

**

**

1. **Edema**

**

Supplementary Fig. 7** Forest plots showing the risk of adverse events following isotonic and hypotonic fluids in hospitalized children
