# Supplementary material for: Efficacy and safety of isotonic versus hypotonic intravenous maintenance fluids in hospitalized children: an updated systematic review and meta-analysis of randomized controlled trials
Source: Pediatr Nephrol. 2023 Jun 26;39(1):57–84. doi: 10.1007/s00467-023-06032-7 (PMC10673968; doi:10.1007/s00467-023-06032-7)
Supplement: Supplementary file 10 — Supplementary file9 (DOCX 4596 KB) [file 467_2023_6032_MOESM10_ESM.docx]

**A**

**
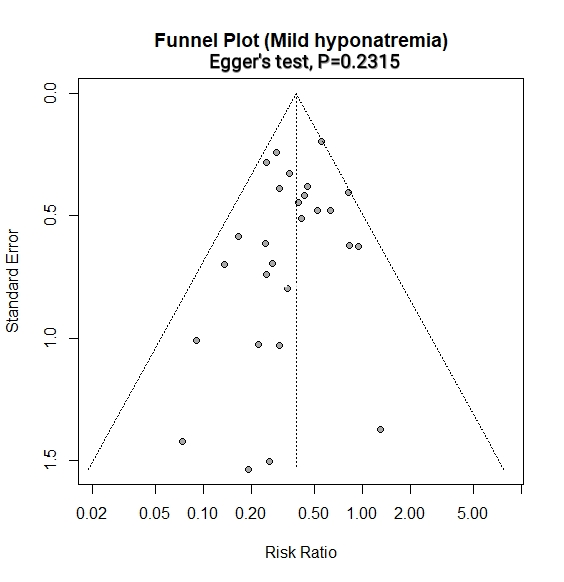
**

**B**

**
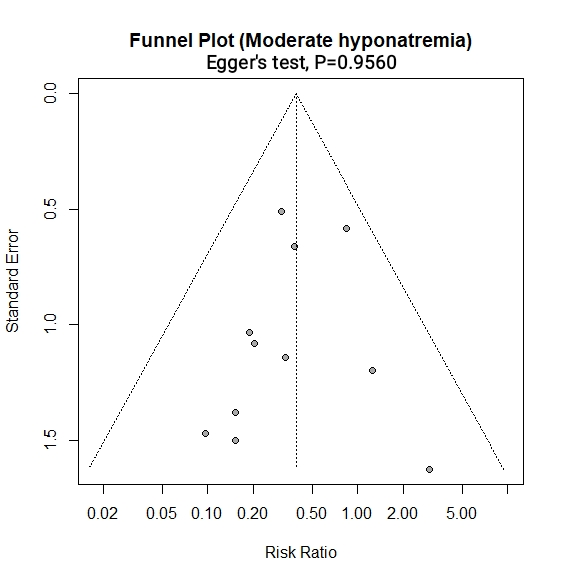
**

**C**

**
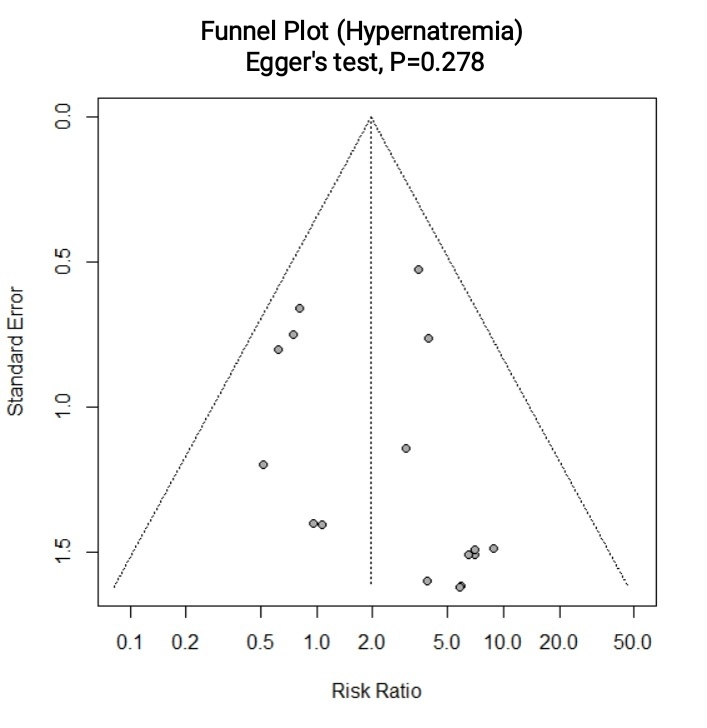
**

**Supplementary Fig. 8** Funnel plots visually representing publication bias assessing risk ratio of A) mild hyponatremia, B) moderate hyponatremia, and C) hypernatremia
