# Supplementary material for: Efficacy and safety of isotonic versus hypotonic intravenous maintenance fluids in hospitalized children: an updated systematic review and meta-analysis of randomized controlled trials
Source: Pediatr Nephrol. 2023 Jun 26;39(1):57–84. doi: 10.1007/s00467-023-06032-7 (PMC10673968; doi:10.1007/s00467-023-06032-7)
Supplement: Supplementary file 11 — Supplementary file10 (DOCX 2558 KB) [file 467_2023_6032_MOESM11_ESM.docx]

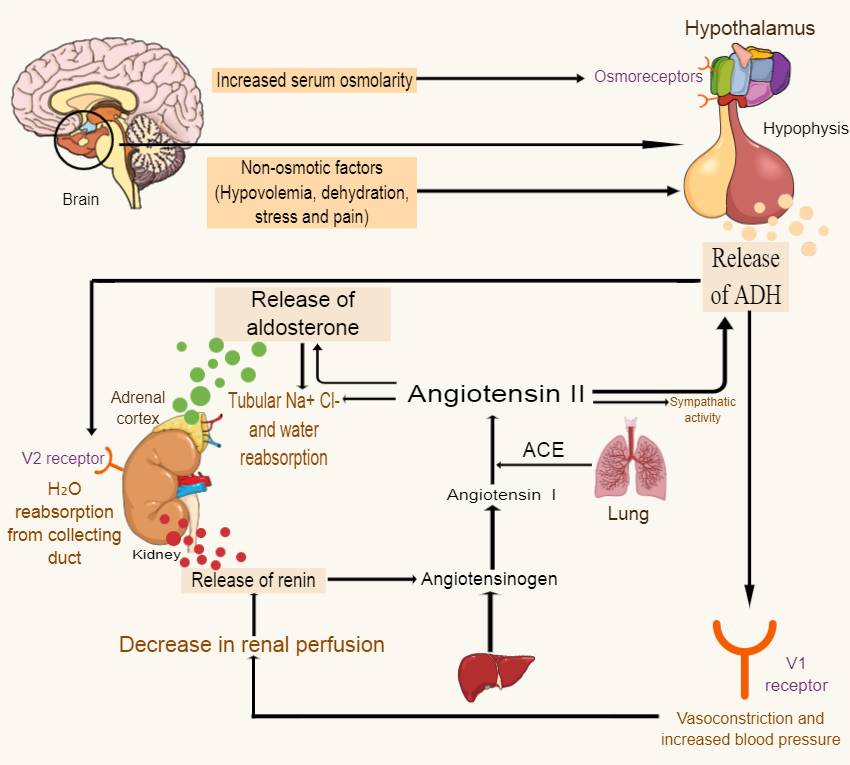


**Supplementary Fig. 9** Illustration of how ADH regulates water balance and sodium homeostasis by different mechanisms. Osmotic and non-osmotic ADH stimuli trigger the release of ADH from the anterior pituitary. ADH acts on V1 receptors, causing vasoconstriction and increased blood pressure, which further decreases renal perfusion and activates the renin-angiotensin-aldosterone system, resulting in increased angiotensin II, which further stimulates sympathetic activity, stimulates more ADH release, and causes salt and water retention either directly or by stimulating the release of aldosterone. In addition, ADH acts on V2 receptors in renal collecting tubules causing water retention. ADH, anti-diuretic hormone; ACE, angiotensin converting enzyme. Created by [mindthegraph.com](https://mindthegraph.com/)
