# Supplementary material for: Efficacy and safety of isotonic versus hypotonic intravenous maintenance fluids in hospitalized children: an updated systematic review and meta-analysis of randomized controlled trials
Source: Pediatr Nephrol. 2023 Jun 26;39(1):57–84. doi: 10.1007/s00467-023-06032-7 (PMC10673968; doi:10.1007/s00467-023-06032-7)
Supplement: Supplementary file 12 — Supplementary file11 (DOCX 18 KB) [file 467_2023_6032_MOESM12_ESM.docx]

**Supplementary Table 1** Search terms and results in different databases

| PubMed | ((Saline OR NaCl OR isotonic OR hypotonic) AND (children OR pediatric OR paediatric OR adolescent OR infant OR newborn) AND (hyponatremia OR hyponatraemia)) | All Fields | 498 |
| --- | --- | --- | --- |
| Web of Science | ((Saline OR NaCl OR isotonic OR hypotonic) AND (children OR pediatric OR paediatric OR adolescent OR infant OR newborn) AND (hyponatremia OR hyponatraemia)) | Topic | 346 |
| Scopus | (("0.9% Saline" OR NaCl OR "Normal Saline" OR "isotonic saline" OR "hypotonic saline") AND (children OR pediatric OR paediatric OR adolescent OR infant OR newborn) AND (hyponatremia OR hyponatraemia)) | Article title, Abstract, Keywords | 192 |
| Cochrane | ((Saline OR NaCl OR isotonic OR hypotonic) AND (children OR pediatric OR paediatric OR adolescent OR infant OR newborn) AND (hyponatremia OR hyponatraemia)) | Title, Abstract, Keyword | 104 |
