# Supplementary material for: Efficacy and safety of isotonic versus hypotonic intravenous maintenance fluids in hospitalized children: an updated systematic review and meta-analysis of randomized controlled trials
Source: Pediatr Nephrol. 2023 Jun 26;39(1):57–84. doi: 10.1007/s00467-023-06032-7 (PMC10673968; doi:10.1007/s00467-023-06032-7)
Supplement: Supplementary file 13 — Supplementary file12 (DOCX 88 KB) [file 467_2023_6032_MOESM13_ESM.docx]

**Supplementary Table 2** Baseline characteristics of patients included in the study

| **Study ID** | **Groups** | **Sample**  **size (n)** | **Age Mean ± SD  or Range** | **Males n (%)** | **Weight (Kg) Mean ± SD** | **Diagnosis** | | **Serum sodium level (mEq/L) Mean ± SD** | **Serum potassium level (mEq/L) Mean ± SD** | **Serum chloride level (mEq/L) Mean ± SD** | **Serum bicarbonate level (mEq/L) Mean ± SD** | **Serum osmolarity (mOSm/L) Mean ± SD** | **Serum creatinine (µmol/L) Mean ± SD** | **Serum urea (mg/dl) Mean ± SD** | **Blood PH Mean ± SD** | **Urinary sodium (mEq/L) Mean ± SD** |
| --- | --- | --- | --- | --- | --- | --- | --- | --- | --- | --- | --- | --- | --- | --- | --- | --- |
|  |  |  |  |  |  | **Medical**  **n (%)** | **Surgical**  **n (%)** |  |  |  |  |  |  |  |  |  |
| Almeida 2015  [1] | 0.9% NaCl | 130 | 49.9 ± 62.5 (months) | 80 (62) | NR | 63 (48.46) | 33 (25.38) | 138.1 ± 4.30 | NR | 103.7 ± 4.50 | 24.8 ± 3.99 | NR | NR | NR | 7.34 ± 0.093 | NR |
|  | 0.45% NaCl | 103 | 41.1 ± 64.4 (months) | 53 (57) | NR | 58 (56.31) | 20 (19.42) | 137.7 ± 3.09 | NR | 102.4 ± 4.14 | 24.9 ± 4.47 | NR | NR | NR | 7.34 ± 0.097 | NR |
| Bagri 2019  [2] | 0.9% NaCl | 75 | 52.92 ± 72.56 (months) | 53 (70.7) | 13.72 ± 10.36 | 75 (100) | 0 | 136.3 ± 4.7 | 4.4 ± 0.7 | 99.1 ± 6.7 | NR | 267.5 ± 12.9 | 45.76 ± 7.63 | 30.9 ± 15.5 | NR | NR |
|  | NaCl 0.45% | 75 | 64.58 ± 80.88 (months) | 54 (72.0) | 13.27 ± 8.16 | 75 (100) | 0 | 135.1 ± 4.4 | 4.4 ± 0.8 | 97.1 ± 5.5 | NR | 267.7 ± 14.9 | 45.76 ± 15.25 | 32.5 ± 12.4 | NR | NR |
| Balasubramanian 2011 [3] | 0.9% saline | 42 | 5.5 ± 1.9 (days) | 23 (55) | NR | 42 (100) | 0 | 142 ± 6 | NR | NR | NR | 294 ± 11 | NR | NR | NR | 50 ± 17 |
|  | 0.2% saline | 42 | 4.9 ± 2 (days) | 23 (55) | NR | 42 (100) | 0 | 143 ± 6 | NR | NR | NR | 295 ± 11 | NR | NR | NR | 49 ± 18 |
| Brazel 1996  [4] | Isotonic saline (Hartman's solution) | 5 | 12.3 – 18.1 (years) | 0 | NR | 0 | 5 (100) | 140.84 ± 1.71 | NR | NR | NR | 290.9 ± 1.22 | NR | NR | NR | NR |
|  | Hypotonic saline (0.3% or 0.18% saline) | 7 | 12.3 – 18.1 (years) | 0 | NR | 0 | 7 (100) | 139.46 ± 2.15 | NR | NR | NR | 290.92 ± 2.38 | NR | NR | NR | NR |
| Chinnasami 2022  [5] | 0.9% saline | 50 | 53.86 ± 47.20 (months) | 32 (64) | NR | 50 (100) | 0 | 141.42 ± 3.45 | 4.27 ± 0.59 | 105.36 ± 3.8 | 17.50 ± 4.3 | NR | 40.42 ± 9.91 | 20.68 ± 6.98 | NR | NR |
|  | Isotonic saline (Plasma-Lyte 148) | 50 | 61.28 ± 48.39 (months) | 29 (58) | NR | 50 (100) | 0 | 137.86 ± 4.41 | 4.10 ± 0.38 | 102.82 ± 4.92 | 18.9 ± 3.69 | NR | 41.18 ± 10.68 | 19.64 ± 6.75 | NR | NR |
|  | 0.18% saline | 50 | 46.51 ± 49.4 (months) | 26 (52) | NR | 50 (100) | 0 | 137.54 ± 4.59 | 4.32 ± 0.53 | 102.54 ± 4.97 | 18.24 ± 3.76 | NR | 38.89 ± 9.91 | 21.08 ± 8.96 | NR | NR |
| Choong 2011  [6] | 0.9% saline | 128 | 9.2 ± 5.5 (years) | 53 (41.4) | 36.0 ± 23.0 | 0 | 128 (100) | 140 ± 2.2 | NR | NR | NR | NR | NR | NR | NR | NR |
|  | 0.45% saline | 130 | 9.2 ± 5.7 (years) | 62 (47.7) | 38.6 ± 26.8 | 0 | 130 (100) | 138 ± 3.2 | NR | NR | NR | NR | NR | NR | NR | NR |
| Coulthard 2012  [7] | Isotonic saline (Hartman's solution) | 41 | 117.2 ± 88.35 (months) | 16 (39) | 34.16 ± 26.12 | 0 | 41 (100) | 139.4 ± 1.5 | 4 ± 0.4 | 105.3 ± 3.0 | 24.1 ± 2.9 | 285.9 ± 3.2 | 44.6 ± 28.4 | NR | NR | NR |
|  | 0.45% saline | 41 | 125.58 ± 74.52 (months) | 23 (56) | 33.06 ± 22.28 | 0 | 41 (100) | 140 ± 2.4 | 4.1 ± 0.4 | 105.4 ± 2.6 | 23.8 ± 3.0 | 288.5 ± 4.7 | 40.6 ± 18.4 | NR | NR | NR |
| Dathan 2021  [8] | NaCl 0.9% | 31 | 4.07 ± 2.33 (days) | 21 (67.7) | 2.58 ± 0.68 | 46 (76.6) | 14 (23.3) | 139.8 ± 2.17 | 4.3 ± 0.54 | NR | NR | 288.32 ± 5.37 | 56.18 ± 16.7 | 26.6 ± 8.7 | NR | NR |
|  | NaCl 0.15% | 29 | 3.45 ± 0.97 (days) | 21 (72.4) | 2.54 ± 0.55 |  |  | 139.8 ± 2.17 | 4.3 ± 0.54 | NR | NR | 288.32 ± 5.37 | 56.18 ± 16.7 | 26.6 ± 8.7 | NR | NR |
| Flores Robles 2015 [9] | 0.3% saline | 49 | 63.5 ± 56.1 (months) | 28 (57.1) | 20.3 ± 13.7 | 35 (71.4) | 14 (28.6) | 135.0 ± 2.8 | 4.0 ± 0.5 | 100.0 ± 4.3 | NR | 277.4 ± 10.6 | 34.4 ± 14.1 | NR | NR | NR |
|  | 0.45% saline | 50 | 54.6 ± 55.9 (months) | 30 (60) | 19.8 ± 16.7 | 36 (72) | 14 (28.0) | 135.8 ± 3.1 | 4.1 ± 0.6 | 101.2 ± 3.1 | NR | 278.0 ± 16.2 | 32.7 ± 11.4 | NR | NR | NR |
|  | 0.9% saline | 52 | 58.8 ± 57.7 (months) | 27 (51) | 21.2 ± 18.9 | 35 (67.3) | 17 (32.7) | 135.8 ± 3.2 | 3.8 ± 0.4 | 100.9 ± 3.1 | NR | 281.4 ± 9.5 | 30.9 ± 11.4 | NR | NR | NR |
| Friedman 2015  [10] | 0.9 % NaCl | 54 | 4.29 ± 3.73 (years) | 28 (51.9) | 16.93 ± 9.22 | 54 (100) | 0 | 138.7 ± 2.5 | NR | NR | NR | NR | NR | NR | NR | NR |
|  | 0.45% NaCl | 56 | 6.15 ± 7.46 (years) | 26 (46.4) | 21.38 ± 19.86 | 56 (100) | 0 | 139.5 ± 2.6 | NR | NR | NR | NR | NR | NR | NR | NR |
| Jorro Baron 2013  [11] | 0.45% NaCl | 32 | 5.57 ± 4.67 (months) | 18 (56.2) | 9.23 ± 8.54 | 29 (85) | 5 (15) | 139.6 ± 3.2 | NR | NR | NR | NR | NR | NR | NR | NR |
|  | 0.9 % NaCl | 31 | 6.07 ± 5.44 (months) | 19 (61.3) | 7.07 ± 3.89 | 30 (93.5) | 2 (6.5) | 139.2 ± 3.5 | NR | NR | NR | NR | NR | NR | NR | NR |
| Raksha 2017  [12] | 0.9% saline | 120 | 1 (month) – 18 (years) | 68 (56.7) | NR | 120 (100) | 0 | 138.53 ± 2.52 | 4.00 ± 0.37 | 101.19 ± 3.24 | NR | NR | NR | NR | NR | NR |
|  | 0.18% saline | 120 | 1 (month) –18 (years) | 66 (55) | NR | 120 (100) | 0 | 138.95 ± 2.84 | 4.93 ± 9.31 | 99.80 ± 9.23 | NR | NR | NR | NR | NR | NR |
| Ramathan 2015  [13] | 0.9% saline | 59 | 2 – 6 (months) | 39 (66) | NR | 59 (100) | 0 | 140.8 ± 5.3 | 4.14 ± 0.4 | NR | NR | NR | 43.32 ± 13.26 | 30.8 ± 10.3 | NR | NR |
|  | 0.18% saline | 60 | 2 – 6 (months) | 40 (66) | NR | 60 (100) | 0 | 147.2 ± 4.99 | 4.43 ± 0.62 | NR | NR | NR | 48.62 ± 15.03 | 28.9 ± 11.4 | NR | NR |
| Ratnjet 2022  [14] | 0.45% saline | 100 | 35.38 ± 58.67 (months) | 61 (61) | NR | 100 (100) | 0 | 136.39 ± 3.35 | NR | NR | 20.33 ± 2.22 | NR | NR | NR | 7.40 ± 0.03 | NR |
|  | 0.9% saline | 100 | 32.77 ± 46.82 (months) | 56 (56) | NR | 100 (100) | 0 | 136.67 ± 3.4 | NR | NR | 20.78 ± 2.76 | NR | NR | NR | 7.40 ± 0.032 | NR |
| Rey 2011  [15] | Hypotonic | 62 | 65.19 ± 74.61 (months) | NR | 22.06 ± 18.75 | 37 (59.5) | 25 (40.3) | 135.9 ± 3.3 | 4.0 ± 0.5 | NR | NR | NR | 38.01 ± 12.38 | 24.8 ± 8.7 | NR | NR |
|  | Isotonic | 63 | 70.71 ± 78.14 (months) | NR | 21.3 ± 18.97 | 31 (49) | 32 (50.8) | 136.0 ± 3.4 | 3.9 ± 0.5 | NR | NR | NR | 37.13 ± 14.14 | 26.3 ± 8.1 | NR | NR |
| Saba 2011  [16] | 0.45% saline | 21 | 9.04 ± 11.77 (years) | 10 (48) | NR | 6 (29) | 15 (71) | 136.64 ± 2.39 | NR | NR | NR | NR | NR | NR | NR | NR |
|  | 0.9% saline | 16 | 8.45 ± 9.35 (years) | 8 (50) | NR | 6 (38) | 10 (62) | 137.46 ± 2.84 | NR | NR | NR | NR | NR | NR | NR | NR |
| Kannan 2010  [17] | 0.9% saline | 58 | 44.48 ± 54.74 (months) | 36 (62) | 13.18 ± 7.98 | 58 (100) | 0 | 138.92 ± 1.09 | NR | NR | NR | NR | NR | NR | NR | NR |
|  | 0.18% saline at standard maintenance | 56 | 44.01 ± 45.12 (months) | 43 (76.78) | 13.81 ± 8.9 | 56 (100) | 0 | 138 ± 0.87 | NR | NR | NR | NR | NR | NR | NR | NR |
|  | 0.18% saline at 2/3 of standard maintenance | 53 | 37.41 ± 42.67 (months) | 35 (66) | 12.65 ± 8.76 | 53 (100) | 0 | 138.91 ± 1.1 | NR | NR | NR | NR | NR | NR | NR | NR |
| Kumar 2020  [18] | 0.9% saline | 84 | 17.76 ± 17.35 (months) | 59 (70) | NR | 84 (100) | 0 | 137.2 ± 2.1 | NR | NR | NR | NR | 53.04 ± 8.84 | 28.3 ± 20.8 | NR | NR |
|  | 0.45% saline | 84 | 15.05 ± 17.73 (months) | 51 (61) | NR | 84 (100) | 0 | 137.9 ± 2.8 | NR | NR | NR | NR | 53.04 ± 8.84 | 25.6 ± 18.4 | NR | NR |
| Lehtiranta 2020  [19] | 0.9% saline | 308 | 4.0 ± 3.1 (years) | 161 (52) | 17 ± 9 | 293 (95) | 15 (5) | 138 ± 2.8 | 4.1 ± 0.4 | NR | 22 ± 4 | NR | NR | NR | 7.4 ± 0.06 | NR |
|  | 0.45% saline | 306 | 4.1 ± 3.1 (years) | 154 (50) | 17 ± 9 | 286 (93) | 20 (7) | 138 ± 2.6 | 4.1 ± 0.5 | NR | 22 ± 3.7 | NR | NR | NR | 7.4 ± 0.06 | NR |
| Mcnab 2015  [20] | 0.9% saline | 338 | 8.2 ± 5.4 (years) | 174 (51) | 31.2 ± 21.2 | 174 (51) | 164 (49) | 137.8 ± 3.1 | NR | NR | NR | NR | NR | NR | NR | NR |
|  | 0.45% saline | 338 | 8.9 ± 5.3 (years) | 172 (51) | 33.2 ± 21 | 190 (46) | 148 (44) | 138.2 ± 3.1 | NR | NR | NR | NR | NR | NR | NR | NR |
| Mierzewska-Schmidt 2015  [21] | Ringer's acetate | 30 | 6.06 ± 2.05 (years) | 24 (80) | 23.1 ± 8.28 | 0 | 30 (100) | 142.57 ± 2.53 | NR | NR | NR | NR | NR | NR | NR | NR |
|  | 0.3% NaCl | 27 | 6.51 ± 2.46 (years) | 16 (57) | 25.8 ± 9.45 | 0 | 27 (100) | 143.3 ± 2.21 | NR | NR | NR | NR | NR | NR | NR | NR |
|  | 5% glucose in water solution | 33 | 6.17 ± 2.07 (years) | 19 (57.58) | 22.7 ± 6.82 | 0 | 33 (100) | 142.3 ± 2.49 | NR | NR | NR | NR | NR | NR | NR | NR |
| Montaana 2008  [22] | Isotonic (NaCl =140 mEQ/L) | 59 | 4.93 ± 6.6 (years) | 35 (59.3) | 18 ± 14.05 | 9 (15.25) | 50 (84.75) | 136.64 ± 3.8 | 4.16 ± 1.14 | NR | NR | NR | NR | NR | NR | NR |
|  | Hypotonic (NaCl <100 mEQ/L) | 63 | 3.67 ± 4.62 (years) | 28 (44.4) | 15.59 ± 13.28 | 10 (15.87) | 53 (84.13) | 136.75 ± 4.02 | 4.23 ± 0.91 | NR | NR | NR | NR | NR | NR | NR |
| Neville 2006  [23] | 0.9% saline | 51 | 2.7 ± 1.5 (years) | 23 (45) | NR | 51 (100) | 0 | 135 ± 3.6 | 4 ± 0.5 | NR | 17.8 ± 3 | 281 ± 8 | 45.9 ± 10.4 | 33.63 ± 11.41 | NR | NR |
|  | 0.45% saline | 51 | 3.1 ± 2 (years) | 30 (59) | NR | 51 (100) | 0 | 136 ± 2.8 | 4 ± 0.5 | NR | 17.9 ± 2.9 | 281 ± 7 | 46.6 ± 12.2 | 30.63 ± 11.41 | NR | NR |
| Neville 2010  [24] | 0.9% saline at standard maintenance rate | 31 | 8.24 ± 3.48 (years) | 14 (45.16) | NR | 0 | 31 (100) | 137 ± 1.9 | NR | 106 ± 1.8 | NR | 286 ± 5 | 44 ± 15 | 18.62 ± 6 | NR | 149 ± 49 |
|  | 0.9% saline at half standard maintenance rate | 31 | 9.06 ± 3.38 (years) | 15 (48.39) | NR | 0 | 31 (100) | 138 ± 1.8 | NR | 104 ± 2.6 | NR | 286 ± 5.2 | 45 ± 12.3 | 19.22 ± 8.41 | NR | 104 ± 58 |
|  | 0.45% saline at standard maintenance rate | 31 | 8.8 ± 3.4 (years) | 17 (54.84) | NR | 0 | 31 (100) | 138 ± 1.6 | NR | 106 ± 2.1 | NR | 286 ± 4.5 | 42 ± 10.8 | 18.62 ± 7.21 | NR | 110 ± 59 |
|  | 0.45% saline at half standard maintenance rate | 31 | 9.57 ± 3.16 (years) | 17 (54.84) | NR | 0 | 31 (100) | 138 ± 1.9 | NR | 105 ± 2.9 | NR | 287 ± 4.3 | 46 ± 12.4 | 21.62 ± 8.41 | NR | 96 ± 37 |
| Pemde 2014  [25] | 0.9% saline | 31 | 26.17 ± 19.59 (months) | NR | 8.9 ± 3.5 | 31 (100) | 0 | 139.03 ± 3.52 | 4.88 ± 0.76 | NR | NR | NR | 46.85 ± 15.03 | 29.39 ±15.18 | NR | NR |
|  | 0.45% saline | 30 | 31.96 ± 20.73 (months) | NR | 10.87 ± 4.88 | 30 (100) | 0 | 139.3 ± 3.28 | 4.58 ± 1.03 | NR | NR | NR | 53.92 ± 15.02 | 32.65 ± 23.17 | NR | NR |
|  | 0.18% saline | 31 | 28.25 ± 21.77 (months) | NR | 9.29 ± 4.29 | 31 (100) | 0 | 138.61 ± 3.60 | 4.77 ± 0.99 | NR | NR | NR | 60.11 ± 22.98 | 33.14 ± 24.41 | NR | NR |
| Omoifo 2018  [26] | Normal saline | 20 | 5.90 ± 3.54 (years) | 13 (65) | 24.60 ± 14.98 | NR | 20 (100) | 132.40 ± 3.70 | 3.90 ± 0.43 | NR | NR | NR | NR | NR | NR | NR |
|  | Isotonic (Ringer lactate) | 20 | 7.00 ± 4.98 (years) | 16 (80) | 26.30 ± 17.30 | NR | 20 (100) | 136.38 ± 6.95 | 3.90 ± 0.61 | NR | NR | NR | NR | NR | NR | NR |
|  | 0.18% saline | 25 | 6.56 ± 3.70 (years) | 18 (72) | 22.77 ± 11.90 | NR | 25 (100) | 136.10 ± 4.68 | 3.86 ± 0.41 | NR | NR | NR | NR | NR | NR | NR |
| Yung 2009  [27] | 0.9% saline at 2/3 of the standard maintenance rate | 13 | 6.14 ± 9.22 (years) | NR | 20.65 ± 23.25 | 2 (16) | 11 (84) | 140 ± 2 | 4.2 ± 0.3 | 108 ± 4 | 22.8 ± 3.7 | 298 ± 18 | 0.05 ± 0.02 | 4.3 ± 1.2 | NR | 80 ±72 |
|  | 0.9% saline at standard maintenance rate | 11 | 13.89 ± 4.33 (years) | NR | 48.78 ± 41.65 | 1 (9) | 10 (91) | 141 ± 2 | 4.1 ± 0.6 | 109 ± 3 | 22.7 ± 3.3 | 299 ± 11 | 0.05 ± 0.02 | 4.2 ± 1.4 | NR | 64 ± 45 |
|  | 0.18% saline at 2/3 of the standard maintenance rate | 15 | 5.03 ± 6.13 (years) | NR | 15.33 ± 10.71 | 7 (47) | 8 (53) | 141 ± 2 | 4.1 ± 0.6 | 107 ± 5 | 20.8 ± 2.5 | 298 ± 8 | 0.04 ± 0.01 | 5.3 ± 3.8 | NR | 99 ± 71 |
|  | 0.18% saline at standard maintenance rate | 11 | 6.94 ± 11.19 (years) | NR | 21.46 ± 24.7 | 3 (28) | 8 (72) | 141 ± 3 | 4.8 ± 1.7 | 108 ± 5.5 | 21.9 ± 5.6 | 299 ± 16 | 0.05 ± 0.03 | 4.9 ± 1.5 | NR | 96 ± 60 |
| Shatabi 2022  [28] | 0.9% saline | 35 | 20.14 ± 10.45 (days) | 20 (57.1) | 2.93 ± 0.375 | 0 | 35 (100) | 140.00 ± 4.03 | 4.25 ± 0.46 | NR | NR | NR | NR | NR | NR | NR |
|  | 0.45% saline | 35 | 15.83 ± 11.21 (days) | 24 (68.6) | 2.85 ± 0.39 | 0 | 35 (100) | 139.86 ± 4.89 | 4.08 ± 0.55 | NR | NR | NR | NR | NR | NR | NR |
| Torres 2019  [29] | 0.9% saline | 155 | 44.7 ± 80.88 (months) | 59 (41.26) | NR | 103 (72.03) | 40 (26.49) | 142.65 ± 5.24 | 4.07 ± 0.97 | NR | NR | NR | NR | NR | 7.4 ± 0.9 | NR |
|  | 0.45% saline | 163 | 45.59 ± 79.34 (months) | 73 (48.34) | NR | 114 (75.5) | 37 (25.87) | 142.7 ± 2.99 | 3.9 ± 0.75 | NR | NR | NR | NR | NR | 7.4 ± 0.07 | NR |
| Shamim 2014  [30] | 0.9% saline | 30 | 53.1 ± 39.5 (months) | 17 (56.7) | NR | 30 (100) | 0 | 135.7 ± 4.2 | 3.9 ± 0.8 | 99.8 ± 6.3 | NR | 291.4 ± 10 | 61.88 ± 7.07 | 39.6 ± 8.6 | 7.36 ± 0.06 | NR |
|  | 0.18% saline | 30 | 54.4 ± 31.7 (months) | 16 (53.3) | NR | 30 (100) | 0 | 136.3 ± 3.5 | 4.1 ± 0.6 | 101.3 ± 6.6 | NR | 293.8 ± 8.5 | 68.06 ± 7.96 | 44.2 ± 9.5 | 7.36 ± 0.06 | NR |
| Omoh 2021  [31] | Isotonic (Ringer lactate) | 25 | 32.52 ± 18.53 (months) | NR | 12.28 ± 5.11 | 0 | 25 (100) | NR | NR | NR | NR | NR | NR | NR | NR | NR |
|  | 0.18% saline | 25 | 28.72 ± 20.3 (months) | NR | 12.10 ± 5.12 | 0 | 25 (100) | NR | NR | NR | NR | NR | NR | NR | NR | NR |
| Valadao 2015  [32] | 0.9% NaCl | 23 | 8.8 ± 3.6 (years) | 13 (43.3) | 35.1 ± 14.0 | 0 | 23 (100) | 135.7 ± 3.3 | 3.9 ± 0.4 | 100.3 ± 2.3 | NR | NR | 53.04 ± 8.84 | NR | NR | NR |
|  | 0.18% NaCl | 27 | 10.3 ± 2.9 (years) | 17 (56.7) | 37.1 ± 12.2 | 0 | 27 (100) | 135.8 ± 2.9 | 3.9 ± 0.4 | 99.0 ± 3.5 | NR | NR | 53.04 ± 8.84 | NR | NR | NR |
| Sherazi 2021  [33] | Isotonic (Ringer lactate) | 78 | 2 ± 1.76 (years) | 43 (55) | NR | 78 (100) | 0 | 137 ± 111.34 | NR | NR | NR | NR | NR | NR | NR | NR |
|  | 0.45% saline | 78 | 2 ± 1.93 (years) | 45 (58) | NR | 78 (100) | 0 | 133 ± 101.09 | NR | NR | NR | NR | NR | NR | NR | NR |

NR: Not reported.

**References**

1. Almeida HI, Mascarenhas MI, Loureiro HC, Abadesso CS, Nunes PS, Moniz MS, MacHado MC (2015) The effect of NaCl 0.9% and NaCl 0.45% on sodium, chloride, and acid-base balance in a PICU population. J Pediatr (Rio J) 91:499–505. https://doi.org/10.1016/j.jped.2014.12.003

2. Bagri NK, Saurabh VK, Basu S, Kumar A (2019) Isotonic v ersus Hypotonic Intravenous Maintenance Fluids in Children: A Randomized Controlled Trial. Indian J Pediatr 86:1011–1016. https://doi.org/10.1007/s12098-019-03011-5

3. Balasubramanian K, Kumar P, Saini SS, Attri SV, Dutta S (2012) Isotonic versus hypotonic fluid supplementation in term neonates with severe hyperbilirubinemia - A double-blind, randomized, controlled trial. Acta Paediatr Int J Paediatr 101:236–241. https://doi.org/10.1111/j.1651-2227.2011.02508.x

4. Brazel PW, McPhee IB (1996) Inappropriate secretion of antidiuretic hormone in postoperative scoliosis patients: The role of fluid management. Spine (Phila. Pa. 1976). 21:724–727

5. Chinnasami B, Manoj P, Reddy K, Dhinakaran R, Chaitanya MSKM (2022) Effect of 0 . 9 % saline in 5 % dextrose , Plasma-Lyte 148 and Isolyte-P used as intravenous maintenance fluids on the electrolyte status of non-critically ill hospitalised children : Results of a prospective randomised open label study. 51:111–118

6. Choong K, Arora S, Cheng J, Farrokhyar F, Reddy D, Thabane L, Walton JM (2011) Hypotonic versus isotonic maintenance fluids after surgery for children: A randomized controlled trial. Pediatrics 128:857–866. https://doi.org/10.1542/peds.2011-0415

7. Coulthard MG, Long DA, Ullman AJ, Ware RS (2012) A randomised controlled trial of Hartmann’s solution versus half normal saline in postoperative paediatric spinal instrumentation and craniotomy patients. Arch Dis Child 97:491–496. https://doi.org/10.1136/archdischild-2011-300221

8. Dathan K, Sundaram M (2021) Comparison of isotonic versus hypotonic intravenous fluid for maintenance fluid therapy in neonates more than or equal to 34 weeks of gestational age – a randomized clinical trial. J Matern Neonatal Med 0:1–8. https://doi.org/10.1080/14767058.2021.1911998

9. Flores Robles CM, Cuello García CA (2016) A prospective trial comparing isotonic with hypotonic maintenance fluids for prevention of hospital-acquired hyponatraemia. Paediatr Int Child Health 36:168–174. https://doi.org/10.1179/2046905515Y.0000000047

10. Friedman JN, Beck CE, Degroot J, Geary DF, Sklansky DJ, Freedman SB (2015) Comparison of isotonic and hypotonic intravenous maintenance fluids: A randomized clinical trial. JAMA Pediatr 169:445–451. https://doi.org/10.1001/jamapediatrics.2014.3809

11. Jorro Baron F (2013) Hypotonic versus isotonic intravenous maintenance fluids in critically ill pediatric patients: a randomized clinical trial. Arch Argent Pediatr 111:281–287. https://doi.org/10.5546/aap.2013.eng.281

12. Raksha SK, Dakshayani B, Premalatha R (2017) Full volume isotonic (0.9%) vs. Two-thirds volume hypotonic (0.18%) intravenous maintenance fluids in preventing hyponatremia in children admitted to pediatric intensive care unit-A randomized controlled study. J Trop Pediatr 63:454–460. https://doi.org/10.1093/tropej/fmx012

13. Ramanathan S, Kumar P, Mishra K, Dutta AK (2016) Isotonic versus Hypotonic Parenteral Maintenance Fluids in Very Severe Pneumonia. Indian J Pediatr 83:27–32. https://doi.org/10.1007/s12098-015-1791-6

14. Kumar Ratnjeet, Pallavi Pallavi, Urmila Jhamb RS (2022) Fluids in Children Admitted With Acute Illness. Pediatr Emer Care 00:436–441. https://doi.org/10.1097/PEC.0000000000002621

15. Rey C, Los-arcos M, Hernández A, Sánchez A, Díaz J (2011) Hypotonic versus isotonic maintenance fluids in critically ill children : a multicenter prospective randomized study. 1138–1143. https://doi.org/10.1111/j.1651-2227.2011.02209.x

16. Saba TG, Fairbairn J, Houghton F, Laforte D, Foster BJ (2011) A randomized controlled trial of isotonic versus hypotonic maintenance intravenous fluids in hospitalized children. BMC Pediatr 11:. https://doi.org/10.1186/1471-2431-11-82

17. Kannan L, Lodha R, Vivekanandhan S, Bagga A, Kabra SK, Kabra M (2010) Intravenous fluid regimen and hyponatraemia among children: A randomized controlled trial. Pediatr Nephrol 25:2303–2309. https://doi.org/10.1007/s00467-010-1600-4

18. Kumar M, Mitra K, Jain R (2020) Isotonic versus hypotonic saline as maintenance intravenous fluid therapy in children under 5 years of age admitted to general paediatric wards: a randomised controlled trial. Paediatr Int Child Health 40:44–49. https://doi.org/10.1080/20469047.2019.1619059

19. Lehtiranta S, Honkila M, Kallio M, Paalanne N, Peltoniemi O, Pokka T, Renko M, Tapiainen T (2021) Risk of Electrolyte Disorders in Acutely Ill Children Receiving Commercially Available Plasmalike Isotonic Fluids: A Randomized Clinical Trial. JAMA Pediatr 175:28–35. https://doi.org/10.1001/jamapediatrics.2020.3383

20. McNab S, Duke T, South M, Babl FE, Lee KJ, Arnup SJ, Young S, Turner H, Davidson A (2015) 140 mmol/L of sodium versus 77 mmol/L of sodium in maintenance intravenous fluid therapy for children in hospital (PIMS): A randomised controlled double-blind trial. Lancet 385:1190–1197. https://doi.org/10.1016/S0140-6736(14)61459-8

21. Mierzewska-Schmidt M (2015) Intraoperative fluid management in children-A comparison of three fluid regimens. Anaesthesiol Intensive Ther 47:125–130. https://doi.org/10.5603/AIT.2015.0012

22. Montañana PÃ, Modesto I Alapont V, Ocón AP, López PO, López Prats JL, Toledo Parreño JD (2008) The use of isotonic fluid as maintenance therapy prevents iatrogenic hyponatremia in pediatrics: A randomized, controlled open study. Pediatr Crit Care Med 9:589–597. https://doi.org/10.1097/PCC.0b013e31818d3192

23. Article O (2006) Isotonic is better than hypotonic saline for intravenous rehydration of children with gastroenteritis: a prospective randomised study. 226–232. https://doi.org/10.1136/adc.2005.084103

24. Neville KA, Hon M, Sandeman DJ, Hon M, Rubinstein A, Henry GM, Mcglynn M, Walker JL (2010) Prevention of Hyponatremia during Maintenance Intravenous Fluid Administration: A Prospective Randomized Study of Fluid Type versus Fluid Rate. J Pediatr 156:313-319.e2. https://doi.org/10.1016/j.jpeds.2009.07.059

25. Pemde HK, Dutta AK, Sodani R, Mishra K (2015) Isotonic Intravenous Maintenance Fluid Reduces Hospital Acquired Hyponatremia in Young Children with Central Nervous System Infections. Indian J Pediatr 82:13–18. https://doi.org/10.1007/s12098-014-1436-1

26. Omoifo CE, Edomwonyi NP, Idogun SE (2018) Incidence of hyponatraemia following the use of three different intravenous fluids in paediatric surgery. African J Paediatr Surg 15:69–72. https://doi.org/10.4103/ajps.AJPS_40_16

27. Yung M, Keeley S (2009) Randomised controlled trial of intravenous maintenance fluids. 45:9–14. https://doi.org/10.1111/j.1440-1754.2007.01254.x

28. Article O (2022) Comparison the Effects of Using Two Methods of Fluid Therapy with Normal Saline or 5% Dextrose in Half Amount of Normal Saline Solution on Blood Glucose and Plasma Electrolytes during and After Neonatal Surgeries: A Randomized Controlled Trial. 79–85. https://doi.org/10.4103/jcn.jcn

29. Torres SF, Iolster T, Schnitzler EJ, Siaba Serrate AJ, Sticco NA, Rivarola MR (2019) Hypotonic and isotonic intravenous maintenance fluids in hospitalised paediatric patients: A randomised controlled trial. BMJ Paediatr Open 3:1–6. https://doi.org/10.1136/bmjpo-2018-000385

30. Shamim A, Afzal K, Manazir Ali S (2014) Safety and efficacy of isotonic (0.9%) vs. hypotonic (0.18%) saline as maintenance intravenous fluids in children: A randomized controlled trial. Indian Pediatr 51:969–974. https://doi.org/10.1007/s13312-014-0542-5

31. Omoh AW, Adedapo SO, Ademola TO (2021) Effect of Intravenous Fluid on Perioperative Plasma Sodium Concentration in Pediatric Surgical Patients. 9:14043–14049. https://doi.org/10.22038/ijp.2020.47988.3875

32. Clara M, Pedro J, Carlos J, Santana B, Celiny P, Garcia R (2015) Comparison of two maintenance electrolyte solutions in children in the postoperative appendectomy period : a randomized , controlled trial ଝ. J Pediatr (Rio J) 1–7. https://doi.org/10.1016/j.jped.2015.01.004

33. Sherazi F, Iqbal S, Naz I, Jadoon AZ, Alam SS (2021) Comparison of the Efficacy of Isotonic Vs Hypotonic Maintenance IV Fluids in Maintaining Normal Na Level in. 44:
