# Supplementary material for: Efficacy and safety of isotonic versus hypotonic intravenous maintenance fluids in hospitalized children: an updated systematic review and meta-analysis of randomized controlled trials
Source: Pediatr Nephrol. 2023 Jun 26;39(1):57–84. doi: 10.1007/s00467-023-06032-7 (PMC10673968; doi:10.1007/s00467-023-06032-7)
Supplement: Supplementary file 14 — Supplementary file13 (DOCX 28 KB) [file 467_2023_6032_MOESM14_ESM.docx]

**Supplementary Table 3** GRADE assessment and the overall certainty of each outcome

| **Certainty assessment** | | | | | | | **№ of patients** | | **Effect** | | **Certainty** | **Importance** |
| --- | --- | --- | --- | --- | --- | --- | --- | --- | --- | --- | --- | --- |
| **No of studies** | **Study design** | **Risk of bias** | **Inconsistency** | **Indirectness** | **Imprecision** | **Other considerations** | **Isotonic Intravenous Maintenance Fluids** | **Hypotonic Intravenous Maintenance Fluids** | **Relative (95% CI)** | **Absolute (95% CI)** |  |  |
| **Mild hyponatremia ≤24 h** | | | | | | | | | | | | |
| 22 | randomized trials | serious^a^ | not serious | not serious | not serious | strong association | 119/1180 (10.1%) | 369/1239 (29.8%) | **RR 0.38** (0.30 to 0.48) | **185 fewer per 1,000** (from 208 fewer to 155 fewer) | ⨁⨁⨁⨁ High | CRITICAL |
| **Mild hyponatremia >24 h** | | | | | | | | | | | | |
| 10 | randomized trials | serious^a^ | not serious | not serious | not serious | strong association | 68/940 (7.2%) | 161/999 (16.1%) | **RR 0.48** (0.37 to 0.62) | **84 fewer per 1,000** (from 102 fewer to 61 fewer) | ⨁⨁⨁⨁ High | CRITICAL |
| **Moderate hyponatremia ≤24 h** | | | | | | | | | | | | |
| 11 | randomized trials | serious^a^ | not serious | not serious | not serious | strong association | 19/718 (2.6%) | 57/769 (7.4%) | **RR 0.40** (0.25 to 0.65) | **44 fewer per 1,000** (from 56 fewer to 26 fewer) | ⨁⨁⨁⨁ High | CRITICAL |
| **Moderate hyponatremia >24 h** | | | | | | | | | | | | |
| 4 | randomized trials | serious^a^ | not serious | not serious | not serious | strong association | 10/168 (6.0%) | 33/221 (14.9%) | **RR 0.40** (0.20 to 0.79) | **90 fewer per 1,000** (from 119 fewer to 31 fewer) | ⨁⨁⨁⨁ High | CRITICAL |
| **Severe hyponatremia ≤24 h** | | | | | | | | | | | | |
| 4 | randomized trials | not serious | not serious | not serious | serious^b^ | none | 1/232 (0.4%) | 6/237 (2.5%) | **RR 0.32** (0.06 to 1.54) | **17 fewer per 1,000** (from 24 fewer to 14 more) | ⨁⨁⨁◯ Moderate | IMPORTANT |
| **Severe hyponatremia >24 h** | | | | | | | | | | | | |
| 3 | randomized trials | not serious | not serious | not serious | not serious | strong association | 2/216 (0.9%) | 13/269 (4.8%) | **RR 0.22** (0.06 to 0.85) | **38 fewer per 1,000** (from 45 fewer to 7 fewer) | ⨁⨁⨁⨁ High | CRITICAL |
| **Hypernatremia ≤ 24 h** | | | | | | | | | | | | |
| 10 | randomized trials | serious^a^ | not serious | not serious | not serious | strong association | 38/618 (6.1%) | 14/661 (2.1%) | **RR 2.44** (1.34 to 4.44) | **30 more per 1,000** (from 7 more to 73 more) | ⨁⨁⨁⨁ High | CRITICAL |
| **Hypernatremia >24 h** | | | | | | | | | | | | |
| 6 | randomized trials | not serious | not serious | not serious | serious^b^ | none | 16/584 (2.7%) | 12/637 (1.9%) | **RR 1.40** (0.56 to 3.49) | **8 more per 1,000** (from 8 fewer to 47 more) | ⨁⨁⨁◯ Moderate | IMPORTANT |
| **Serum sodium ≤24 h** | | | | | | | | | | | | |
| 27 | randomized trials | serious^a^ | serious^c^ | not serious | not serious | none | 1790 | 1713 | - | MD **2.53 lower** (3.29 lower to 1.76 lower) | ⨁⨁◯◯ Low | NOT IMPORTANT |
| **Serum sodium >24 h** | | | | | | | | | | | | |
| 10 | randomized trials | serious^a^ | serious^c^ | not serious | serious^b^ | none | 674 | 708 | - | MD **1.39 lower** (3.1 lower to 0.32 higher) | ⨁◯◯◯ Very low | NOT IMPORTANT |
| **Serum osmolarity ≤24 h** | | | | | | | | | | | | |
| 9 | randomized trials | serious^a^ | serious^c^ | not serious | not serious | none | 267 | 224 | - | MD **5.73 lower** (10.22 lower to 1.23 lower) | ⨁⨁◯◯ Low | NOT IMPORTANT |
| **Serum osmolarity >24 h** | | | | | | | | | | | | |
| 3 | randomized trials | serious^a^ | serious^c^ | not serious | serious^b^ | none | 111 | 109 | - | MD **9.04 lower** (23.73 lower to 5.65 higher) | ⨁◯◯◯ Very low | NOT IMPORTANT |
| **Serum chloride ≤24 h** | | | | | | | | | | | | |
| 9 | randomized trials | serious^a^ | serious^c^ | not serious | not serious | none | 519 | 492 | - | MD **1.84 lower** (2.97 lower to 0.72 lower) | ⨁⨁◯◯ Low | NOT IMPORTANT |
| **Serum chloride >24 h** | | | | | | | | | | | | |
| 3 | randomized trials | serious^d^ | serious^c^ | not serious | serious^b^ | none | 154 | 204 | - | MD **1.54 lower** (5.31 lower to 2.23 higher) | ⨁◯◯◯ Very low | NOT IMPORTANT |
| **Serum potassium ≤24 h** | | | | | | | | | | | | |
| 11 | randomized trials | serious^a^ | serious^c^ | not serious | not serious | none | 761 | 799 | - | MD **0.07 higher** (0.24 lower to 0.37 higher) | ⨁⨁◯◯ Low | NOT IMPORTANT |
| **Serum potassium >24 h** | | | | | | | | | | | | |
| 5 | randomized trials | serious^a^ | serious^c^ | not serious | not serious | none | 535 | 487 | - | MD **0.14 higher** (0.39 lower to 0.68 higher) | ⨁⨁◯◯ Low | NOT IMPORTANT |
| **Serum creatinine ≤24 h** | | | | | | | | | | | | |
| 8 | randomized trials | not serious | not serious | not serious | serious^b^ | none | 229 | 283 | - | MD **0.82 higher** (1.8 lower to 3.44 higher) | ⨁⨁⨁◯ Moderate | IMPORTANT |
| **Serum creatinine >24 h** | | | | | | | | | | | | |
| 3 | randomized trials | not serious | not serious | not serious | serious^b^ | none | 227 | 181 | - | MD **0.38 lower** (4.52 lower to 3.76 higher) | ⨁⨁⨁◯ Moderate | IMPORTANT |
| **Blood sugar** | | | | | | | | | | | | |
| 4 | randomized trials | serious^a^ | not serious | serious^e^ | serious^b^ | none | 208 | 256 | - | MD **1.96 higher** (3.56 lower to 7.48 higher) | ⨁◯◯◯ Very low | NOT IMPORTANT |
| **Urinary sodium** | | | | | | | | | | | | |
| 6 | randomized trials | serious^d^ | not serious | not serious | not serious | none | 119 | 123 | - | MD **37.07 lower** (47.53 lower to 26.61 lower) | ⨁⨁⨁◯ Moderate | IMPORTANT |
| **Length of hospital stay** | | | | | | | | | | | | |
| 6 | randomized trials | serious^d^ | not serious | serious^e^ | not serious | none | 662 | 714 | - | MD **0.07 lower** (0.66 lower to 0.51 higher) | ⨁⨁◯◯ Low | NOT IMPORTANT |
| **Blood PH** | | | | | | | | | | | | |
| 3 | randomized trials | serious^a^ | serious^c^ | serious^e^ | not serious | none | 303 | 284 | - | MD **0.05 lower** (0.08 lower to 0.02 lower) | ⨁◯◯◯ Very low | NOT IMPORTANT |
| **Death** | | | | | | | | | | | | |
| 7 | randomized trials | serious^a^ | not serious | serious^e^ | serious^b^ | none | 17/681 (2.5%) | 13/732 (1.8%) | **RR 1.48** (0.72 to 3.06) | **9 more per 1,000** (from 5 fewer to 37 more) | ⨁◯◯◯ Very low | NOT IMPORTANT |
| **Edema** | | | | | | | | | | | | |
| 7 | randomized trials | not serious | not serious | serious^e^ | serious^b^ | none | 34/755 (4.5%) | 20/800 (2.5%) | **RR 1.52** (0.88 to 2.62) | **13 more per 1,000** (from 3 fewer to 41 more) | ⨁⨁◯◯ Low | NOT IMPORTANT |
| **Hypertension** | | | | | | | | | | | | |
| 5 | randomized trials | serious^a^ | not serious | serious^e^ | serious^b^ | none | 9/302 (3.0%) | 11/358 (3.1%) | **RR 0.92** (0.40 to 2.13) | **2 fewer per 1,000** (from 18 fewer to 35 more) | ⨁◯◯◯ Very low | NOT IMPORTANT |
| **seizures** | | | | | | | | | | | | |
| 6 | randomized trials | serious^a^ | not serious | serious^e^ | serious^b^ | none | 2/915 (0.2%) | 8/1011 (0.8%) | **RR 0.45** (0.08 to 2.67) | **4 fewer per 1,000** (from 7 fewer to 13 more) | ⨁◯◯◯ Very low | NOT IMPORTANT |

**CI:** confidence interval; **MD:** mean difference; **RR:** risk ratio

#### Explanations

a. Some Studies at higher risk of bias

b. Wide CI

c. Heterogeneity >50%

d. Most studies are uncertain.

e. not related to intravenous maintenance fluids.
